# Dynamic changes in O-GlcNAcylation regulate osteoclast differentiation and bone loss via nucleoporin 153

**DOI:** 10.1038/s41413-022-00218-9

**Published:** 2022-07-26

**Authors:** Yi-Nan Li, Chih-Wei Chen, Thuong Trinh-Minh, Honglin Zhu, Alexandru-Emil Matei, Andrea-Hermina Györfi, Frederic Kuwert, Philipp Hubel, Xiao Ding, Cuong Tran Manh, Xiaohan Xu, Christoph Liebel, Vladyslav Fedorchenko, Ruifang Liang, Kaiyue Huang, Jens Pfannstiel, Min-Chuan Huang, Neng-Yu Lin, Andreas Ramming, Georg Schett, Jörg H. W. Distler

**Affiliations:** 1 Department of Internal Medicine 3 – Rheumatology and Immunology, Friedrich Alexander University Erlangen-Nürnberg and Universitätsklinikum Erlangen, Erlangen, Germany; 2 Deutsches Zentrum für Immuntherapie, Friedrich Alexander University Erlangen-Nuremberg and Universitaetsklinikum Erlangen, Erlangen, Germany; 3 Department of Rheumatology, Xiangya Hospital, Central South University, Changsha, Hunan China; 4 Core Facility Hohenheim, University of Hohenheim, Stuttgart, Germany; 5 Graduate Institute of Anatomy and Cell biology, National Taiwan University College of Medicine, Taipei, Taiwan

**Keywords:** Bone, Diseases

## Abstract

Bone mass is maintained by the balance between osteoclast-induced bone resorption and osteoblast-triggered bone formation. In inflammatory arthritis such as rheumatoid arthritis (RA), however, increased osteoclast differentiation and activity skew this balance resulting in progressive bone loss. O-GlcNAcylation is a posttranslational modification with attachment of a single O-linked β-D-N-acetylglucosamine (O-GlcNAc) residue to serine or threonine residues of target proteins. Although O-GlcNAcylation is one of the most common protein modifications, its role in bone homeostasis has not been systematically investigated. We demonstrate that dynamic changes in O-GlcNAcylation are required for osteoclastogenesis. Increased O-GlcNAcylation promotes osteoclast differentiation during the early stages, whereas its downregulation is required for osteoclast maturation. At the molecular level, O-GlcNAcylation affects several pathways including oxidative phosphorylation and cell-cell fusion. TNFα fosters the dynamic regulation of O-GlcNAcylation to promote osteoclastogenesis in inflammatory arthritis. Targeted pharmaceutical or genetic inhibition of O-GlcNAc transferase (OGT) or O-GlcNAcase (OGA) arrests osteoclast differentiation during early stages of differentiation and during later maturation, respectively, and ameliorates bone loss in experimental arthritis. Knockdown of NUP153, an O-GlcNAcylation target, has similar effects as OGT inhibition and inhibits osteoclastogenesis. These findings highlight an important role of O-GlcNAcylation in osteoclastogenesis and may offer the potential to therapeutically interfere with pathologic bone resorption.

## Introduction

Rheumatoid arthritis (RA) is the most common chronic inflammatory joint disease affecting up to 1% of the adult population. RA causes progressive joint destruction and leads to severe disability and reduced quality of life^1^. Under physiologic conditions, bone mass is maintained by constant remodeling with a balance between osteoclast-induced bone resorption and osteoblast-triggered bone formation. However, in RA, this balance is shifted towards bone resorption based on increased osteoclast differentiation, resulting in local and systemic bone loss^2^.

Osteoclasts are macrophage polykaryons specialized on bone resorption^3^. Macrophage colony-stimulating factor (M-CSF) and receptor activator of nuclear factor-kB (NF-κB) ligand (RANKL) are key-regulators of osteoclastogenesis^4–6^. Proinflammatory cytokines such as tumor necrosis factor alpha (TNFα) can further amplify osteoclast differentiation^7–9^. TNFα is a key player in the pathogenesis of RA^10^. It is expressed abundantly in the synovium of inflamed joints and stimulates osteoclastogenesis. This is exemplified by the phenotype of mice overexpressing human TNFα (hTNFα tg) that exhibit increased numbers of osteoclasts and develop destructive arthritis, which resembles RA in humans^11^. Moreover, targeted inhibition of TNFα ameliorates inflammation and bone loss in patients with RA, has greatly improved the prognosis of RA patients, and is still the mainstay of RA treatment to date^12–14^.

O-GlcNAcylation involves covalent attachment of a single O-linked β-D-N-acetylglucosamine (O-GlcNAc) residue to serine or threonine residues of proteins^15^. O-GlcNAcylation thereby differs fundamentally from other forms of glycosylation that result in the attachment of large, complex and often branched glycosyl-residues to target sites^16^. The level of O-GlcNAcylation is regulated by only two enzymes: O-GlcNAc transferase (OGT), which is the only enzyme capable of adding O-GlcNAc to target proteins, and O-GlcNAcase (OGA), which is essentially required to remove O-GlcNAc^17^. O-GlcNAcylation is one of the most common protein modifications^18^, but one only recently begins to appreciate its regulatory role in cellular homeostasis. O-GlcNAcylation can alter the susceptibility of target proteins for other post-translational modifications, their subcellular localization, the association with binding partners, and affect protein half-life^19,20^. Via these mechanisms, O-GlcNAc can regulate central cellular events, such as transcription, translation, intracellular trafficking, and differentiation^17^. Deregulation of O-GlcNAcylation has been implicated into the pathogenesis of chronic human diseases such as diabetes, cardiovascular diseases, neurodegeneration, and cancer^21^. Of particular interest, O-GlcNAcylation can modulate inflammatory responses^22,23^. We thus hypothesized that aberrant O-GlcNAcylation may promote an inflammatory milieu that stimulates osteoclastogenesis in arthritis.

In the present study, we characterized the role of O-GlcNAcylation in osteoclastogenesis. We demonstrate a dynamic regulation of O-GlcNAcylation during osteoclastogenesis. Targeted inhibition of this dynamic balance in O-GlcNAcylation affects key regulators of osteoclastogenesis such as cytokine signaling, oxidative phosphorylation, organization of the actin cytoskeleton, and cell-cell fusion and strongly inhibits osteoclastogenesis, in particular in the presence of TNFα and in arthritis. Moreover, pharmaceutical inhibition of O-GlcNAcase or knockout of OGT in the monocyte/macrophage lineage ameliorates inflammation as well as local and systemic bone loss in preclinical models of RA.

## Results

### Dynamic regulation of O-GlcNAcylation during osteoclastogenesis

**Fig. 1:**
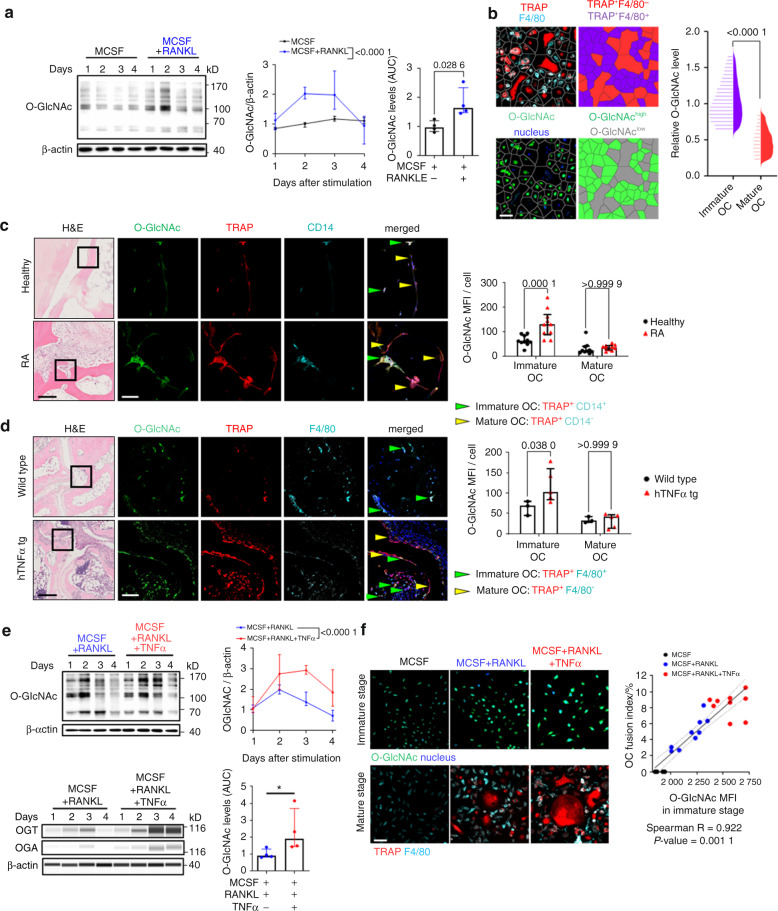
Dynamic regulation of protein O-GlcNacylation during osteoclastogenesis. **a** Western blot analysis of protein O-GlcNAcylation during osteoclastogenesis in vitro (*n* = 4 per group). Representative images and quantification of the changes in the levels of O-GlcNAc normalized to β-actin over time are shown. **b** Representative Voronoi-tessellated cellomics images of in vitro osteoclastogenesis and cellomics analysis of O-GlcNAcylation levels in immature and mature osteoclasts (OC) (*n* = 21025 cells in total). Representative histology and confocal microscopic images of O-GlcNAc, TRAP, and CD14 triple stainings in bone tissues from healthy donors and RA patients (**c**) or wildtype and hTNFα tg mice (**d**). Semi-automated, blinded quantification of O-GlcNAcylation in immature and mature OC in synovial tissue sections (*n* = 10 sections per group). Immature OC (green) and mature OC (yellow) are marked with arrows. **e** Representative blots of Western blots and ProteinSimple Wes immunoassay of O-GlcNAcylation, OGT, and OGA expression (*n* = 4 per group). **f** Representative cellomics images of protein O-GlcNAcylation in immature OC and its correlation (Spearman) with osteoclastogenesis. The OC fusion index represents the proportion of the nuclei in mature OCs (*n* = 9 per group). Bar graphs are shown as median ± IQR. Linear regression and its 95% confidence interval (dashed line) are presented in the correlation plots (**f**). Horizontal scale bars are represented as 50 µm in IF images or 200 µm in H&E images. Statistical significance was determined by Mann-Whitney U-test (**a, b, e**) or two-way ANOVA (**c**, **d**). *P*-values and Spearman’s ρ are shown in the graphs. AUC area under the curve, OC osteoclast, MFI mean fluorescence intensity

We first analyzed the levels of O-GlcNAcylation during the differentiation of osteoclast precursors into mature osteoclasts in vitro. O-GlcNAcylation progressively increased during the early differentiation phase of osteoclastogenesis and peaked at around day 2. Thereafter, O-GlcNAc levels progressively decreased in the maturation process with lowest levels in mature multinucleated osteoclasts (Fig. 1a). Consistently, cellomics analysis showed that immature TRAP and F4/80 double-positive (TRAP^**+**^F4/80^**+**^) osteoclasts had much higher levels of O-GlcNAc compared to TRAP-positive, F4/80 negative (TRAP^**+**^F4/80^**−**^) mature osteoclasts (Fig. 1b).

We next analyzed the levels of O-GlcNAcylation in immature and mature osteoclasts in synovial tissues of patients with RA and matched healthy individuals. Immunofluorescence staining for O-GlcNAc demonstrated very high levels of O-GlcNAcylation in immature TRAP^**+**^CD14^**+**^ osteoclasts of RA patients and low levels in mature, multinucleated TRAP^**+**^CD14^**−**^ osteoclasts. In healthy individuals, immature osteoclasts also stained for O-GlcNAc. However, confocal microscopic analysis with semi-automated, blinded quantification showed that the levels of O-GlcNAc in immature osteoclasts were lower as compared to those in RA patients (Fig. 1c). Similar findings were observed in mice with transgenic overexpression of human TNFα (hTNFα tg mice): TRAP^**+**^F4/80^**+**^ immature osteoclasts expressed very high levels of O-GlcNAcylation and decreased in TRAP^**+**^F4/80^**−**^ mature multinucleated osteoclasts. The levels of O-GlcNAcylation in TRAP^**+**^F4/80^**+**^ immature osteoclast were higher in hTNFα tg mice than in non-transgenic littermates (Fig. 1d).

Together, these findings highlight dynamic O-GlcNAcylation during osteoclastogenesis and suggest that the inflammatory milieu in arthritis enhances O-GlcNAcylation. Consistent with this hypothesis, costimulation of osteoclast precursors with TNFα and RANKL increased the expression of OGT and OGA with higher ratios of OGT/OGA compared to RANKL only, and induced in higher peak levels of O-GlcNAc without limiting its downregulation at later stages (Fig. 1e). Indeed, cellomics analysis revealed that TNFα intensified the upregulation of O-GlcNAc in immature osteoclasts with comparable levels of O-GlcNAc in mature osteoclasts incubated with or without TNFα (Fig. S1). Furthermore, the increase in the peak levels of O-GlcNAcylation correlated with enhanced osteoclast differentiation in the presence of TNFα as shown by cellomics analysis (Fig. 1f).

### Targeting OGT and early-stage increase in O-GlcNAcylation inhibits osteoclastogenesis

**Fig. 2:**
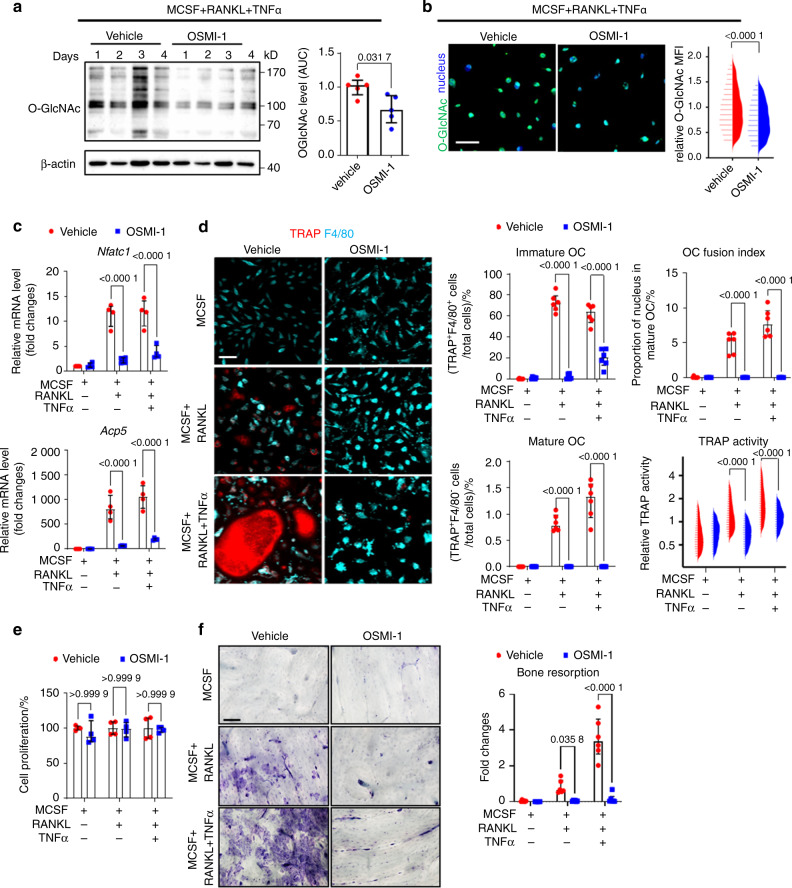
Pharmacological inhibition of OGT arrests osteoclastogenesis at early stages of differentiation. Western blot analysis (**a**), representative images of high-content imaging, and cellomics analysis (**b**) of O-GlcNAcylation during in vitro osteoclastogenesis in the presence of TNFα and with or without OSMI-1 (*n* = 5 per group for western blot; *n* = 11 759 cells in total for cellomics). **c** Levels of osteoclast-related genes analyzed by real-time PCR after OSMI-1 treatment (*n* = 4 per group). **d** Representative images of high-content imaging and the cellomics analysis of the proportions of immature and mature osteoclasts, the OC fusion index, and TRAP enzyme activity (*n* = 102 666 cells in total). **e** Proliferation assay (*n* = 4 per group). **f** In vitro bone resorption assay with OSMI-1 treatment (*n* = 6 per group). All bar graphs are shown as median ± IQR. Horizontal scale bars are represented as 50 µm in IF images, 100 µm in bone resorption assay. Statistical significance was determined by Mann–Whitney U-test (**a**, **b**) or two-way ANOVA (**c–f**). *P*-values are shown in the graphs. AUC area under the curve, MFI mean fluorescence intensity, OC osteoclast

To investigate the functional relevance of these dynamic changes in O-GlcNAcylation for osteoclast differentiation, we inhibited OGT during early stages of osteoclastogenesis using the selective OGT inhibitor OSMI-1^24^. Inhibition of OGT prevented the upregulation of O-GlcNAcylation during the early phase of osteoclastogenesis (Fig. 2a, b) which impaired the transcription of *Nfatc1*, a master regulator of osteoclastogenesis^25–27^, and of *Acp5*, which encodes for the osteoclast marker TRAP^28,29^ (Fig. 2c). OGT inhibition impaired osteoclastogenesis with decreased numbers of immature and mature osteoclasts, reduced OC fusion index, and decreased TRAP activity (Fig. 2d). In contrast, OGT inhibition did not impair the proliferation of osteoclast precursors, indicating a specific effect on osteoclast differentiation and maturation (Fig. 2e). The inhibitory effects of OSMI-1 on osteoclastogenesis translated into impaired capacity for bone resorption in in vitro assays (Fig. 2f).

To confirm these findings on the genetic level, we knocked out OGT in the monocytes lineage. OGT knockout cells and control cells were isolated from the bone marrow of *Ogt*^*ΔLsyM*^ mice (mice with two conditional alleles of OGT (OGT^fl/fl^) and LysM-Cre^30^ expression) and OGT^fl/fl^ littermates without LysM-Cre, respectively. OGT knockout reduced the levels of O-GlcNAcylation (Fig. S2a, b). OGT knockout cells had reduced levels of osteoclast-related genes *Nfatc1* and *Acp5* (Fig. S2c) and demonstrated impaired potential for osteoclast differentiation in vitro with reduced numbers of immature and mature osteoclasts, decreased OC fusion, and lower TRAP activity (Fig. S2e), but without notable changes in proliferation (Fig. S2e). OGT knockout cells also showed impaired capacity for bone resorption in in vitro assays (Fig. S2f). Together, these results demonstrate that the upregulation of O-GlcNAcylation during early stages of differentiation is essential for osteoclastogenesis.

### Targeting OGA and late-stage decrease of O-GlcNAcylation inhibits osteoclastogenesis

**Fig. 3:**
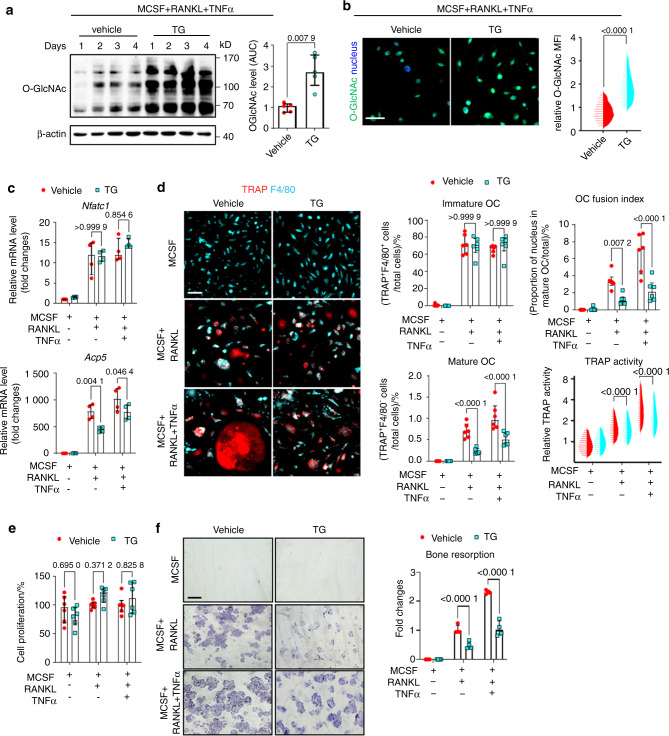
Pharmacological inhibition of OGA impairs osteoclast maturation. Western blot analysis (**a**), representative images of high-content imaging, and cellomics analysis (**b**) of O-GlcNAcylation during in vitro osteoclastogenesis in the presence of TNFα and with or without Thiamet-G (TG) (*n* = 5 per group for western blot; *n* = 17 973 cells in total for cellomics). **c** mRNA levels of osteoclast-related genes (*n* = 4 per group). **d** Representative images of high-content imaging and cellomics analysis of the proportions of immature and mature OC, OC fusion index, and TRAP enzyme activity (*n* = 142 807 cells in total). **e** Proliferation assay (*n* = 6 per group). **f** In vitro bone resorption assay (*n* = 4 per group). All bar graphs are shown as median ± IQR. Horizontal scale bars are represented as 50 µm in IF images, 100 µm in bone resorption assay. Statistical significance was determined by Mann-Whitney U-test (**a**, **b**) or two-way ANOVA (**c–f**). *P*-values are shown in the graphs. AUC area under the curve, MFI mean fluorescence intensity, OC osteoclast

We next investigated the functional relevance of the downregulation of O-GlcNAcylation in later stages of osteoclastogenesis. We therefore inhibited OGA using the selective OGA inhibitor Thiamet-G^31^. Inhibition of OGA prevented the decrease of O-GlcNAcylation in the maturation stage of osteoclastogenesis (Fig. 3a, b). This translated into functional arrest of osteoclastogenesis in the later stages, as shown by reduced mRNA levels of *Acp5*, but unchanged levels of *Nfatc1*, which is upregulated in early stages of osteoclastogenesis (Fig. 3c). Impaired downregulation of O-GlcNAcylation in later stages of osteoclastogenesis blocked maturation of osteoclasts with reduced numbers of multinucleated mature osteoclasts, decreased OC fusion index and TRAP activity, but did not affect proliferation (Fig. 3d, e). Consistent with the kinetics of O-GlcNAcylation with early increases and later downregulation, the number of immature osteoclast precursors was not reduced by OGA inhibition. Thiamet-G reduced bone degradation in in vitro resorption assays (Fig. 3f). In all experiments, modulation of O-GlcNAcylation had particularly pronounced inhibitory effects on osteoclastogenesis in the presence of TNFα with stronger effects as compared to osteoclastogenesis in the presence of RANKL only (Fig. 3). These findings demonstrate that downregulation of O-GlcNAcylation in later stages of osteoclastogenesis is functionally required for osteoclast maturation.

Next, we evaluated the outcome of osteoclastogenesis, when OSMI-1 and Thiamet-G were applied only during early stages of osteoclast differentiation. Two days of OSMI-1 treatment at the early stages is sufficient to block the mature osteoclast formation. However, early-stage OGA inhibition has limited effects in osteoclastogenesis (first two days; Fig. S3). Under those experimental conditions, only OSMI-1, but not Thiamet-G inhibited osteoclastogenesis.

### Targeting O-GlcNAcylation by inhibition of OGT ameliorates inflammatory bone loss

**Fig. 4:**
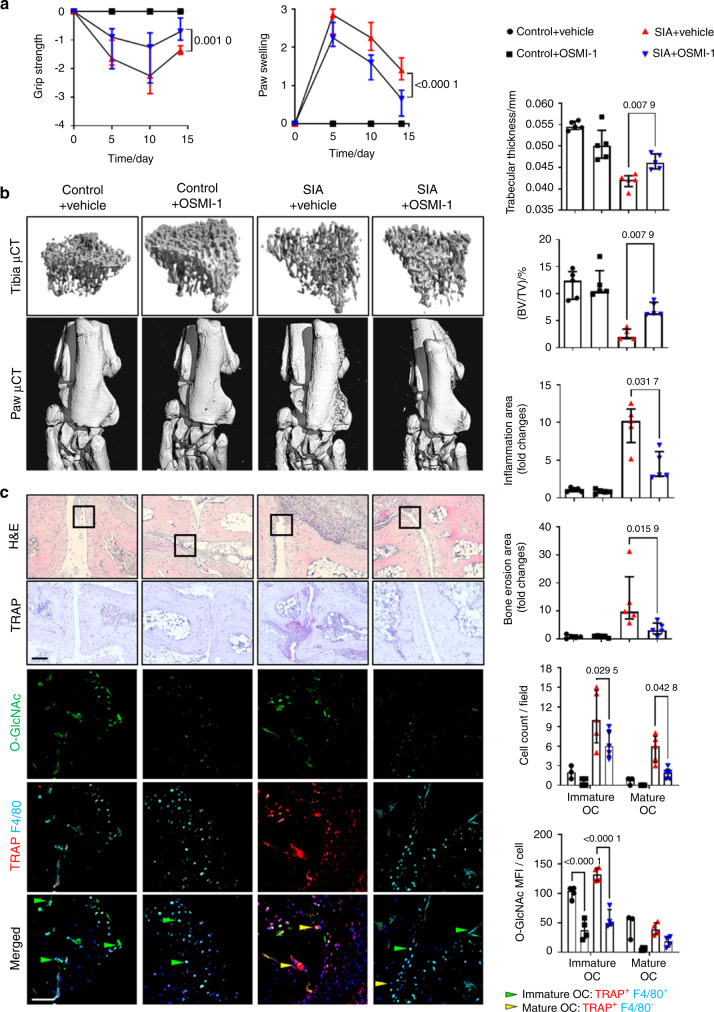
Pharmacological inhibition of OGT ameliorates bone loss in serum-induced arthritis. **a** Grip strength and joint swelling in mice serum-induced arthritis (SIA) treated with OSMI-1. **b** Representative images and quantification of microcomputed tomography scans of bone tissue and analysis of the tibial bone structure. **c** Representative histological and confocal microscopy images of the tarsus with semi-automated, blinded quantification of H&E staining, TRAP staining, and O-GlcNAcylation. (*n* ≥ 4 for all groups). All results are presented as median ± IQR. Horizontal scale bars are represented as 50 µm in IF images, 100 µm in histological staining. Statistical significance was determined by two-way ANOVA (**a, c**) or Mann–Whitney U-test (**b**). *P*-values are shown in the graphs. MFI mean fluorescence intensity, OC osteoclast

We next aimed to confirm the critical role of dynamic changes in O-GlcNAcylation for osteoclastogenesis in vivo. We first targeted the increase in O-GlcNAcylation in early stages of osteoclastogenesis by treatment of mice with serum-induced arthritis (SIA)^32^ with OSMI-1. OSMI-1 treatment mitigated clinical symptoms of arthritis caused by the transfer of autoantibody-containing serum such as reduced grip strength and paw swelling (Fig. 4a). Treatment with OSMI-1 also attenuated bone erosions in the paws as shown by the µCT. OSMI-1 ameliorated not only local bone erosion but also systemic osteoporosis, as shown by quantification of bone volume density in the tibia bones (Fig. 4b). Histological analysis demonstrated less inflammation, reduced bone erosions, and decreased numbers of both, immature and mature osteoclasts by OSMI-1 treatment. Immunofluorescence further demonstrated reduced O-GlcNAcylation levels in OSMI-1 treated mice (Fig. 4c).

We confirmed these findings by LysM-Cre driven knockout of OGT in osteoclast precursor cells in hTNFα tg mice^30^ using bone marrow transplantation. Knockout of OGT in osteoclast precursors prevented inflammation-induced osteoclastogenesis, ameliorated local and systemic bone loss, and reduced joint inflammation (Fig. S4a, b). Consistent with the findings in OSMI-1 treated mice, OGT knockout in hTNFα tg mice abrogated the increase in O-GlcNAcylation and the accumulation of immature and mature osteoclasts in arthritic mice (Fig. S4c).

A recent study showed that O-GlcNAcylation could affect immune responses in macrophages^33^. To exclude that the observed effects on osteoclastogenesis in arthritis models are mediated indirectly by modulation of inflammation, we next tested the role of O-GlcNAcylation in a model with less inflammatory involvement, the ovariectomy-induced osteoporosis (OVX). Indeed, OGT knockdown in monocytic cells did not change leukocyte infiltration into the synovium in the OVX model. However, consistent with the findings in the hTNFα tg and SIA models, OGT knockout in osteoclasts precursors mitigates OVX-induced bone loss and reduces the number of mature osteoclasts (Fig. S5).

Together, these findings demonstrate that the upregulation of O-GlcNAcylation in early stages of differentiation is essential for osteoclastogenesis and osteoclast-mediated bone resorption in inflammatory and non-inflammatory models of bone loss.

### Inhibition of the downregulation of O-GlcNAcylation reduces osteoclastogenesis in experimental arthritis

**Fig. 5:**
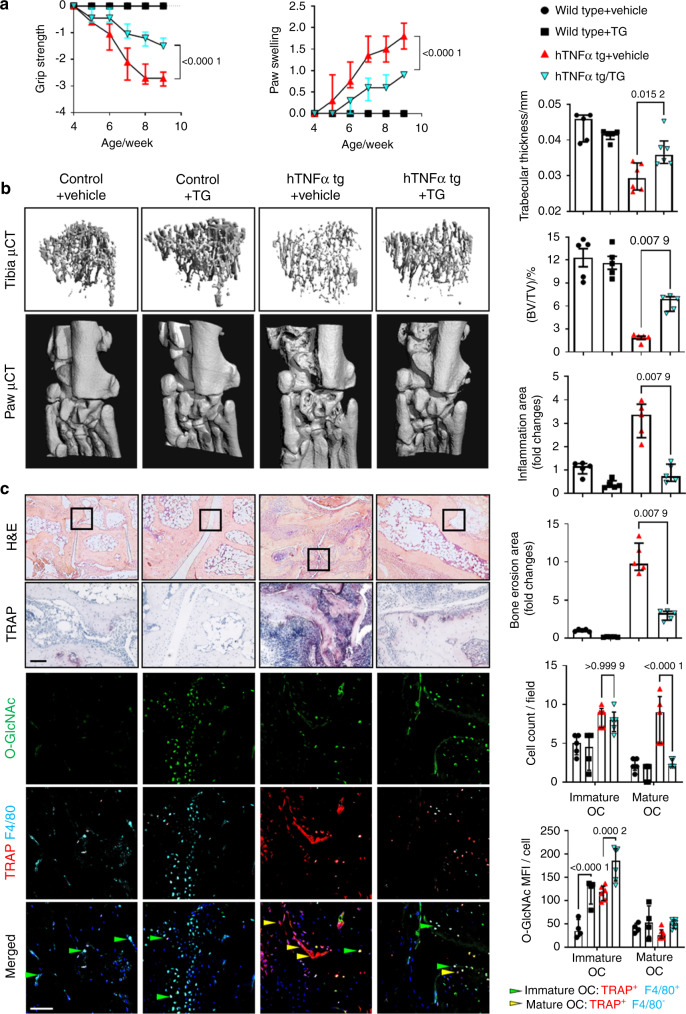
Pharmacological inhibition of OGA ameliorates bone loss in human TNFα transgene mice. **a** Grip strength and joint swelling in hTNFα tg mice treated with Thiamet-G (TG). **b** Images and quantification of microcomputed tomography scans of bone tissue and analysis of the tibial bone structure. **c** Representative histological and confocal images of the tarsus with semi-automated, blinded quantification of H&E staining, TRAP staining, and O-GlcNAcylation. (*n* ≥ 5 for all groups). All results are presented as median ± IQR. Horizontal scale bars are represented as 50 µm in IF images, 100 µm in histological staining. Statistical significance was determined by two-way ANOVA (**a**, **c**) or Mann–Whitney U-test (**b**). *P*-values are shown in the graphs. MFI mean fluorescence intensity, OC osteoclast

We next targeted the downregulation of O-GlcNAcylation in late stages of osteoclastogenesis by inhibition of OGA with Thiamet-G in hTNFα tg mice. Treatment with Thiamet-G ameliorated loss of grip strength and paw swelling (Fig. 5a), reduced local and systemic bone loss with decreased local erosions and systemic osteoporosis (Fig. 5b). Treatment with Thiamet-G also reduced synovial inflammation, increased the levels of O-GlcNAcylation, and reduced the formation of mature osteoclasts without affecting the number of immature osteoclasts (Fig. 5c).

We also analyzed the effects of Thiamet-G in the SIA model. Treatment with Thiamet-G reduced clinical signs of arthritis, ameliorated local and systemic bone loss, and decreased the numbers of mature osteoclasts without reducing the number of immature osteoclasts (Fig. S6).

Together, these data highlight that dynamic regulation of O-GlcNAcylation is critically required for osteoclastogenesis and osteoclast-mediated bone loss.

### Differential, stage-specific transcriptional effects of OGT- and OGA-inhibition

**Fig. 6:**
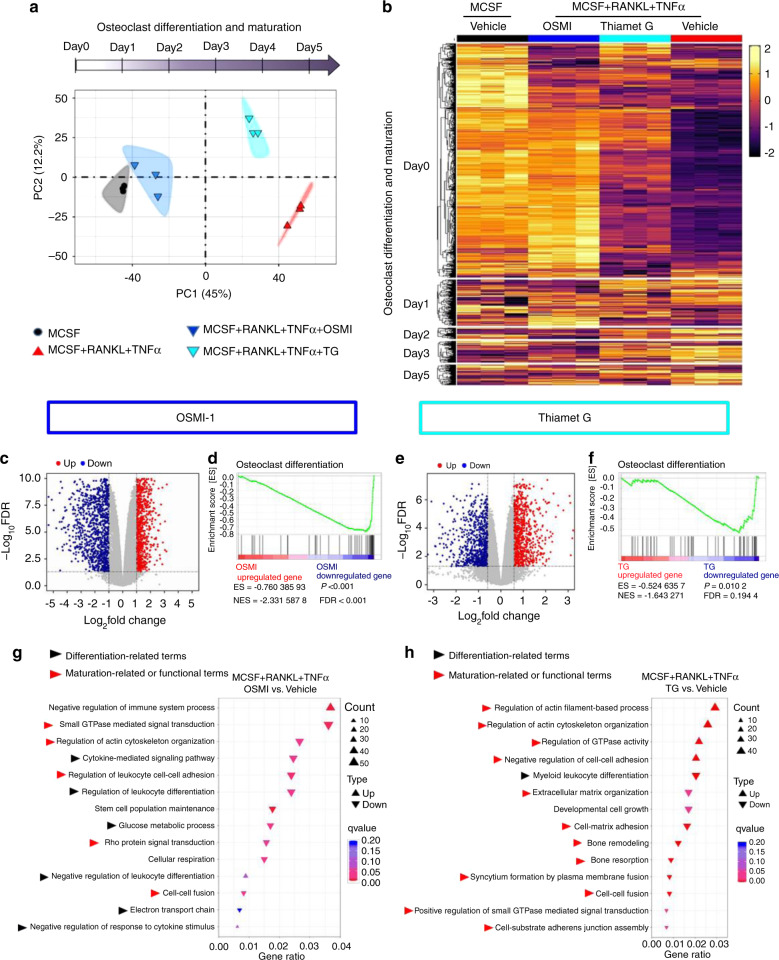
Differential transcriptomic effects of OGT and OGA inhibition during osteoclastogenesis. RNASeq of osteoclast precursor cells with inhibition of OGT by OSMI-1 or inhibition of OGA by Thiamet-G of osteoclastogenesis with respective controls, *n* = 3 for each condition. **a** PCA analysis demonstrating an arrest at different stages by OSMI-1 and Thiamet-G. **b** Expression heatmap of genes implicated in osteoclastogenesis by integration of publically available transcriptome data from different stages of osteoclastogenesis (GSE138324)^34^. **OSMI-1 treatment:** **c** Volcano plot of DEGs. Expression of each gene is plotted as log-fold change of expression ratio with respect to controls; upregulated and downregulated genes are represented by red and green dots, respectively. **d** GSEA enrichment plot for osteoclast differentiation. **g** Geneset functional analysis plots of significantly enriched Gene Ontology (GO) biological processes related to osteoclastogenesis. Upward and downward triangles indicate the processes enriched in up- and down-regulated genes, respectively. **Thiamet-G treatment:** **e** Volcano plot of DEGs. **f** GSEA enrichment plot for osteoclast differentiation. **h** Geneset functional analysis plots of the significantly enriched GO biological processes related to osteoclastogenesis

We next aimed to characterize the molecular mechanisms by which O-GlcNAc regulates osteoclastogenesis. We, therefore, performed RNA sequencing of osteoclast precursors incubated with M-CSF, RANKL, and TNFα with inhibition of OGT at early stages by OSMI-1, with inhibition of OGA at later stages by Thiamet-G, and controls with M-CSF, RANKL, TNFα, and vehicle or with M-CSF and vehicle only. Principal component analysis (PCA) demonstrated that OSMI-1 inhibited osteoclastogenesis at very early stages with transcriptional profiles most closely related to precursors incubated with M-CSF only. In contrast, Thiamet-G arrested osteoclastogenesis at later stages with more similarities to cells incubated to M-CSF, RANKL, and TNFα (Fig. 6a). To further characterize the effects at different stages, we generated a gene set containing genes regulated at different stages of osteoclastogenesis from publically available datasets^34,35^. Expression heatmaps of the gene set further highlight that OSMI-1 and Thiamet-G exert stage-specific effects and that the most osteoclast-related genes are regulated by O-GlcNAcylation (Fig. 6b and Fig. S7).

After application of cutoffs compared to controls, we identified 1865 differentially expressed genes (DEGs) in OSMI-1 treated cells and 1565 DEGs in Thiamet-G treated cells (Fig. 6c, e). Conistent with the potent inhibitory effects on osteoclastogenesis in vitro and in vivo, geneset enrichment analysis (GSEA) demonstrated negative enrichment scores (“de-enrichment”) for genes related to osteoclast differentiation^36^ for OSMI-1 and Thiamet-G (Fig. 6d, f). Using gene functional analysis for Gene Ontology (GO) biological processes, we found profound enrichment of categories relevant for osteoclastogenesis for OSMI-1 and Thiamet-G. However, the individual categories demonstrated differences and OSMI-1 affected in particular categories required for early steps of osteoclast differentiation such as cytokine signaling, leukocyte differentiation and metabolic adaptation (Fig. 6g), whereas Thiamet-G regulated in particular categories implicated in later maturation stages such as cell-cell fusion, rearrangement of the actin cytoskeleton, integrin-mediated cellular adhesion and bone remodelling (Fig. 6h). GSEA pathway enrichment analysis further confirmed the regulation of multiple categories implicated in different stages of osteoclastogenesis by OSMI-1 and Thiamet-G (Fig. S8a, b).

Moreover, our data obtained from OSMI-1 and Thiamet-G treated murine osteoclast precursors both showed distinct, but highly significant association patterns with considerable enrichment scores for genes deregulated in human macrophages obtained from synovial fluid of inflamed joints from patients with RA (Fig. S8c, d). These results demonstrate that O-GlcNAcylation regulates the transcription of core genes and pathways required for inflammation-induced osteoclastogenesis. We next aimed to unravel upstream transcription regulators are capable of regulating the expression of O-GlcNAc-dependent DEGs. Using Ingenuity Pathway Analysis (IPA) with thresholds of |activation z-score|>2 and *P*-value of overlap <0.001, we identified 11 potential transcription regulators of the DEGs in OSMI-1 treated cells (Fig. S9a). Since O-GlcNAcylation is a post-translational modification, we were particularly interested in the regulators without transcriptional changes upon OSMI-1 treatment. These included several well-known transcriptional regulators of osteoclastogenesis (Fig. S9b). Amongst those, MYC (which was recently identified as a key regulator of osteoclastogenesis^37,38^) was proposed to regulate the largest subset of OSMI-DEGs (108 genes) and demonstrated as the most significant target in the two analyses for motif enrichment on OSMI-DEGs (Fig. S9c). GSEA analysis demonstrated downregulation of MYC target genes in osteoclast precursors upon OGT inhibition (Fig. S9d). We matched our RNA-Seq results obtained from osteoclast precursors with OGT inhibition to a dataset of osteoclast precursors with MYC knockout^38^. We observed a 78.6% overlap in the enriched GO biological process (Fig. S9e). These results support our hypothesis on the role of MYC in O-GlcNAc-dependent osteoclastogenesis.

### Inhibition of the O-GlcNAc target nucleoporin 153 (NUP153) disrupts osteoclastogenesis

We next aimed to identify proteins that are differentially O-GlcNAcylated during early stages of osteoclastogenesis using mass spectrometry as an unbiased screening approach. Incubation of osteoclast precursor cells with RANKL and TNFα for 3 days induced O-GlcNAcylation of functionally heterogeneous target proteins including the nuclear pore component NUP153, the MTDH (also known as Metadherin, LYRIC, or astrocyte elevated gene-1 protein (AEG-1)) as a regulator of RNA splicing^39^, IFI207 as a transcriptional regulator of innate immune responses^40^ and RBM27, involved in mRNA decay^41^. Most pronounced differences were observed for NUP153, with 7.7 fold increased upon RANKL and TNFα costimulation compared to controls.

To provide evidence for a functional role of those O-GlcNAcylated proteins in osteoclastogenesis, we evaluated the potential of these O-GlcNAcylated proteins to regulate the O-GlcNAc-driven transcriptome. Therefore, we integrated mass spectrometry findings with the results of our RNASeq experiments. Using IPA, we predicted the capability of the O-GlcNAcylated proteins detected by mass spectrometry to serve as upstream regulators for DEGs identified by RNASeq in OSMI-1 treated samples. IPA proposed interactions between NUP153 and the transcription factor MYC, which had been predicted as the most potent upstream regulator of O-GlcNAc-DEGs in our RNASeq experiments. NUP153 has also been shown to regulate the nuclear transport of MYC^42^. This suggests a model, in which O-GlcNAcylation-induced changes in the activity of NUP153 may modify the nuclear activity of these transcription factors to regulate the expression of osteoclastogenic genes. Based on its most pronounced differences in O-GlcNAcylation levels and its potential to regulate target genes of O-GlcNAcylation, we thus focused on NUP153 as an effector for O-GlcNAcylation-regulated osteoclastogenesis for our further studies (Fig. S10).

**Fig. 7:**
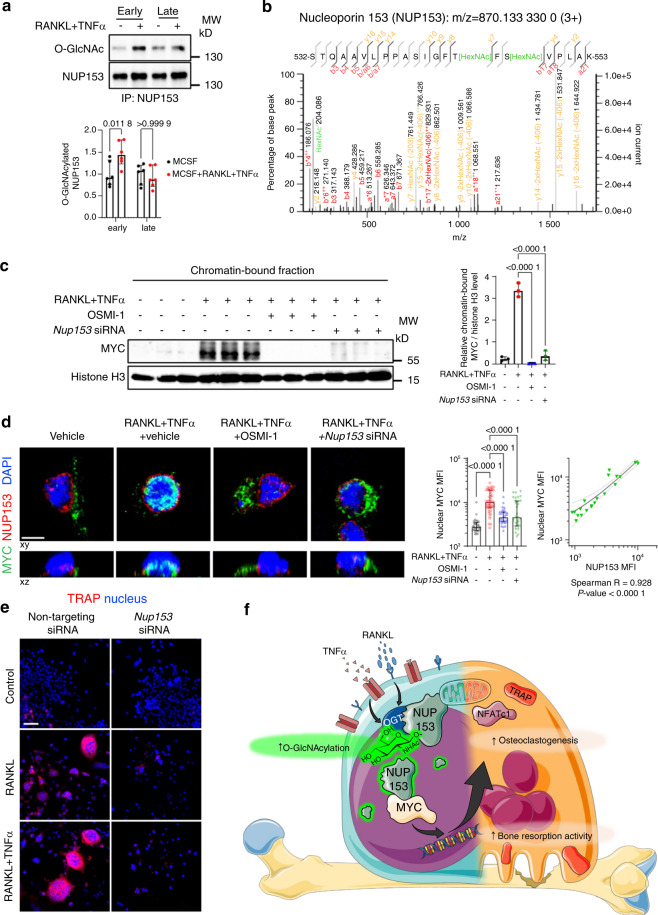
O-GlcNAcylation target NUP153 is essential for MYC nuclear accumulation and osteoclastogenesis. **a** Levels of O-GlcNAcylation on NUP153 in early and late stages of osteoclastogenesis by immunoprecipitation (*n* = 6 per group). **b** MS2 spectrum of O-GlcNAcylated peptide in NUP153 from RANKL and TNFα costimulated RAW264.7 cells. **c** Western blot analysis for chromatin-bound MYC in the cells with *Nup153* knockdown or OSMI-1 (*n* = 3 replicates). **d** Representative immunofluorescence images of confocal microscopy and quantification of the median MYC intensity within the nuclear volume in the cells treated with OSMI-1 and *Nup153* siRNA (*n* ≥ 30 cells per group). Spearman correlation analysis on the intensities of MYC and NUP153 in cells treated with *Nup153* siRNA (*n* = 20 cells). Unless specified, cells were treated with vehicle and non-targeting siRNA. **e** Representative images for TRAP staining on cells transfected with *Nup153* siRNA. **f** Conceptual scheme of the functional role of O-GlcNAcylation modulation of NUP153 during osteoclastogenesis. The illustration was created by using images from Servier Medical Art (http://smart.servier.com/) and Scheng23 (https://commons.wikimedia.org/wiki/File:O-GlcNAc_cycling-1.png), licensed under CC BY 3.0 (https://creativecommons.org/licenses/by/3.0/) and CC BY-SA 4.0 licenses (https://creativecommons.org/licenses/by-sa/4.0/), respectively. Bar graphs are shown as median ± IQR. Horizontal scale bars represent 5 µm (**d**) or 50 µm (**e**) in IF images. Statistical significance was determined by two-way (**a**) or one-way ANOVA (**c**, **d**). *P*-values and Spearman’s ρ are presented in the graphs. Linear regression and its 95% confidence interval (dashed line) are presented in the correlation plots (**d**). MFI, median fluorescence intensity

We first confirmed increased O-GlcNAcylation of NUP153 during the early stages of RANKL/TNFα-induced osteoclastogenesis by immunoprecipitation. Consistent with global O-GlcNAc dynamics, the O-GlcNAcylation of NUP153 decreased at later stages of osteoclastogenesis (Fig. 7a). Further analyses of the mass spectrometry data demonstrated that O-GlcNAcylation was only detected at T546 and S548 of NUP153 in RANKL and TNFα costimulated precursors, but not at other putative sites (Fig. 7b).

Consistent with the proposed mode of action, knockdown of *Nup153* decreased the nuclear accumulation of MYC upon RANKL and TNFα costimulation (Fig. 7c, d, and Fig. S11a). To verify the functional role of NUP153 in osteoclastogenesis, we knockdown *Nup153* during osteoclast differentiation by siRNA. We found that *Nup153* knockdown decreased *Nfatc1* and *Acp5* mRNA levels (Fig. S11b). Knockdown of *Nup153* also lowered the percentage of immature and mature osteoclast, OC fusion index, and TRAP activity (Fig. 7e and Fig. S11c), thus mimicking the findings of OSMI treatment during early stages of osteoclastogenesis. In vitro bone resorption assays demonstrated impaired pit formation in cells treated with *Nup153* siRNA compared to control siRNA (Fig. S11d). Together, these results demonstrate that the O-GlcNAc target NUP153 regulates the nuclear translocation and thus the transcriptional activity of MYC to control the expression of osteoclast differentiation genes and promote osteoclast differentiation in vitro (Fig. 7f).

## Discussion

We demonstrate that osteoclast differentiation requires a dynamic regulation of O-GlcNAcylation with increasing levels during early stage and progressive decline to baseline levels in late stage of osteoclastogenesis. Interference with the upregulation of O-GlcNAcylation during early stage induces an arrest in osteoclast differentiation characterized by profound transcriptional inhibition of cytokine signaling and prevention of metabolic adaptation in differentiating cells. Inhibition of the downregulation of O-GlcNAc in late stages of osteoclastogenesis by inhibition of OGA also blocks osteoclastogenesis. The mechanisms accounting for those later effects are different and include inhibition of rearrangement of the actin cytoskeleton and of osteoclast fusion. Inhibition of OGT in early stages of differentiation is sufficient to impair the formation of mature osteoclasts, whereas inhibition of OGA during the early stages has no significant effects on osteoclast numbers. These findings demonstrate that O-GlcNAcylation controls multiple different cellular processes to regulate osteoclastogenesis in a stage-dependent manner.

The upregulation of OGT mRNA in osteoclastogenic conditions was blocked by p38 inhibition, but not by inhibition of Erk, Src, cJUN/AP1. This result points to a p38-dependent regulation of O-GlcNAcylation in early phases of osteoclast differentiation. However, we did not observe shifts in the ratio of OGT and OGA levels in favor of OGA in late stages of osteoclastogenesis. The regulation of OGA may thus occur on the levels of its enzymatic activity rather on the level of transcription or translation. Indeed, several factors have been shown to affect OGA activity and stability, such as the interaction with OGT at Ser405^43^, Tax protein binding^44^, nuclear localization upon nucleolar stress^45^, or cellular redox balance^46^. Cellular redox environment and nucleolar function have been shown to be involved in osteoclastogenesis^47,48^. However, elucidation of the detailed regulation of OGA activity in osteoclasts requires further studies.

The induction of *Nfatc1* by RANKL can be separated into two phases: an initial induction by NFATc2 and NF-κB and later auto-amplification of NFATc1 by self-amplifying mechanisms^26^. Our transcriptome data showed that OGT inhibition blocked *Nfatc1* induction without affecting the expression of p65, p50, and IκBα. This results provides preliminary evidence that O-GlcNAcylation rather modulate the auto-amplification of *Nfatc1* than its initial NF-κB-dependent induction. However, the mechanism of O-GlcNAc-regulated *Nfatc1* expression requires further studies.

Our study shows that TNFα, a key mediator of inflammation in arthritis, fosters the dynamic regulation of O-GlcNAcylation during osteoclastogenesis. TNFα increases the expression levels of OGT and OGA to promote enhanced osteoclastogenesis in early stages without affecting its downregulation in later stages. Other proinflammatory cytokines such as IL-1 or metabolic adaptions may also contribute to this early upregulation of O-GlcNAcylation^49,50^. However, the mechanisms coordinating the biphasic regulation of O-GlcNAcylation with downregulation during later stages of osteoclastogenesis require further studies. Consistent with this regulatory effect of TNFα on O-GlcNAcylation, inhibition of O-GlcNAcylation was particularly effective when osteoclast precursors were cultured in the presence of TNFα. Together, these findings provide evidence that TNFα regulates O-GlcNAylation to promote excessive osteoclastogenesis in inflammatory arthritis including RA in humans.

Our study highlights that interference with the dynamic regulation of O-GlcNAcylation can be employed as a strategy to target aberrant osteoclastogenesis. Pharmacologic inhibition of OGT by OSMI-1 or monocyte-specific knockout of OGT inhibits osteoclastogenesis by blocking early transcriptional events of osteoclastogenesis. Although the molecular mechanisms of action are different, targeting of OGA by Thiamet-G arrests osteoclast differentiation and fusions, thereby preventing the formation of mature osteoclasts. Both treatments strongly reduce the accumulation of mature osteoclasts with prevention of local bone erosions in the inflamed joints as well as amelioration of systemic, inflammation-induced bone loss. O-GlcNAcylation has also been reported to regulate osteoblastic differentiation of the MC3T3-E1 cell line^51^. However, differentiation of MC3T3-E1 cells requires no dynamic regulation, but constantly increasing levels of O-GlcNAcylation. While Thiamet-G may thus in part increase bone density by fostering osteoblast differentiation, targeting of OGT would rather inhibit osteoblast function. Potential effects on osteoblasts are thus insufficient to explain the findings obtained with OGT and OGA modulation in our arthritis models.

Mechanistically, O-GlcNAcylation may simultaneously regulate several core pathways of osteoclastogenesis in a NUP153-dependent manner. IPA suggested MYC as the most potent upstream regulator of OGT target genes. A recent study proposed MYC as a central component driving the regulatory gene network for osteoclast-lineage commitment^37^. MYC has also been shown to trigger metabolic reprogramming during osteoclast differentiation^38^. Our GO and GSEA analysis showed metabolism-related pathways are highly enriched in the OGT inhibited transcriptome. The unaltered transcription levels of MYC upon OGT inhibition together with the absence in mass spectrometry analysis indicate an indirect regulation of their activity via another target protein. Indeed, our newly identified O-GlcNAcylation target NUP153 is capable of interacting with MYC^42^. NUP153 is known to control nucleoplasmic trafficking and regulate gene expression by direct binding with chromatin and transcription factors^52^. Indeed, we demonstrate that knockdown of *Nup153* impairs nuclear translocation and chromatin binding of MYC. Consistently, osteoclast precursor cells were arrested at similar differentiation status with comparable functional deficits upon OGT inhibition and upon *Nup153* knockdown. Data mining of RNASeq results from osteoclast precursors with MYC knockout also supports an O-GlcNAc-dependent regulation of MYC. O-GlcNAcylation is also known to regulate transcriptional events by direct modification of transcription factors. Examples include O-GlcNAc-induced changes in the stability of c-Jun in HEK 293T cells^53^, as well as O-GlcNAc-induced changes in the transcriptional activities of NF-κB and NFATc1 in lymphocytes^54^. Further studies are required to analyze the potential roles of other O-GlcNAcylated proteins in osteoclastogenesis and how they may synergize with NUP153 to regulate osteoclastogenesis. Our IPA analysis on RNA-Seq data of osteoclast precursors treated with Thiamet-G indicated deregulation of several pathways relevant for osteoclast maturation, such as CREBBP, IRF/STAT, and MAFB. Haploinsufficient CREBBP mice displayed osteoporotic phenotype with hyper-active osteoclastogenesis^55^. Enhanced osteoclast fusion was observed in IRF1- or STAT1-knockout cells^56^. MAFB is also known as a negative regulator for osteoclast differentiation^57^. The role of O-GlcNAcylation for the regulation of these pathways requires further experimental validation. Although further experiments are required for confirmation, these findings indicate that the effects of O-GlcNAcylation on osteoclast differentiation might be mediated by O-GlcNAcylation-induced differences in NUP153 activity with deregulation of the nuclear access of MYC.

In addition to impaired osteoclastogenesis and preservation of bone integrity, we observed anti-inflammatory effects in hTNFα tg and SIA models with pharmacological inhibition and also with monocyte-specific inactivation of OGT. O-GlcNAcylation has previously been shown to stimulate the release of pro-inflammatory cytokines from synovial fibroblasts in NfκB-dependent manner^22^. The anti-inflammatory effects of pharmacological modulation of O-GlcNAc may thus at least in part dependent on impaired cytokine release from synovial fibroblasts. However, the finding that targeted inactivation of OGT in monocytic cells also strongly ameliorates synovial inflammation highlights that monocyte-lineage cells are also targeted by the anti-inflammatory effects of O-GlcNAc in experimental arthritis. This conclusion is further supported by our findings that multiple GO- and GESA-terms related to cytokine signaling and inflammation are regulated by O-GlcNAcylation in osteoclast precursors. Although pro-inflammatory in our murine models of arthritis, the role of O-GlcNAcylation in inflammatory conditions is complex and cell type dependent^58,59^. Both pro- and anti-inflammatory effects have also been reported in certain settings, e.g., by MAVS-dependent enhancement of anti-viral immunity^60^, by RIPK3-dependent suppression of necroptosis in sepsis^61^, and involvement in both Treg and Th17 development^15,62^. The effects of targeted modulation of O-GlcNAcylation on inflammation in arthritis thus deserve further studies. However, we also provide evidence that the regulatory effects of O-GlcNAcylation are not exclusively mediated indirectly by modulation of inflammation: (1) interference with the dynamic regulation of O-GlcNAc also blocked osteoclastogenesis in vitro; (2) inactivation of OGT also ameliorated osteoclast formation and bone loss in the OVX model, as a less inflammatory model of bone loss.

In summary, we demonstrate that O-GlcNAcylation regulates several critical pathways of osteoclastogenesis. Initial increases in O-GlcNAc promote cytokine signaling and metabolic adaptation to promote differentiation of osteoclast precursors, while downregulation of O-GlcNAc in later stages stimulates rearrangement of the actin cytoskeleton and enhances cell-cell fusion to promote osteoclast maturation. Interference with the dynamic regulation of O-GlcNAcylation by different pharmacologic or genetic approaches blocks osteoclast differentiation and ameliorates local and systemic bone loss in arthritis.

## Material and methods

### Patient recruitment

Synovial tissues were obtained from ten RA patients with a disease duration of 1.5–27 years and seropositive, erosive RA. All RA patients fulfilled the 2010 American College of Rheumatology (ACR) classification criteria for RA^63^. Tissue samples from matched healthy donors were included for the comparison.

### Mice

Wildtype male C57BL/6JRj mice were supplied by Janvier Labs (Saint-Berthevin, France). Transgenic mice that overexpressed human TNFα (hTNFα tg)^64,65^, *Ogt*-floxed^66^, and *LysM*-Cre^30,65^ (The Jackson Laboratory, Bar Harbor, ME, USA) have been described previously. To knockout *Ogt* in myeloid lineage cells, we generated *Ogt*^*ΔLysM*^ mice by cross-breeding *Ogt*-floxed and *LysM*-Cre. We used *Ogt*-floxed mice as littermate control for all the *Ogt* knockout experiments.

### Bone marrow isolation and in vitro osteoclastogenesis

In vitro osteoclastogenesis was performed as previously described^38,67^ with minor modifications. In brief, bone marrow cells from 8 to 12-week old wildtype, *Ogt*-floxed, or *Ogt*^*ΔlysM*^ mice were harvested by flushing the tibia and femur of mice with α-MEM (Gibco, New York, USA), followed by the lysis of red blood cells with RBC lysis buffer (Biolegend, San Diego, CA, USA). Afterwards, cells were cultured in in 10% FBS α-MEM overnight. The non-adherent cells were then seeded at the density of 2.5 × 10^5^ cells per mL and expanded with 25 ng·mL^−1^ M-CSF (R&D Systems, Wiesbaden-Nordenstadt, Germany) for 3 days. Osteoclastogenesis was induced with 25 ng·mL^−1^ M-CSF, 50 ng·mL^−1^ RANKL (Peprotech, Hamburg, Germany) with or without 20 ng·mL^−1^ TNFα (R&D Systems) for 4 days. The culture medium was changed every 2 days.

### Cellomics and TRAP activity for in vitro osteoclastogenesis

Cells were fixed in 4% formaldehyde and permeabilized in 0.2% Triton X-100 after 2 days (immature phase) or 4 days (mature phase) of in vitro osteoclastogenesis. After blocking with 5% horse serum in 2% BSA/PBS, cells were incubated with antibodies against F4/80 (Bio-Rad, Feldkirchen, Germany; clone Cl: A3-1) and O-GlcNAc (Invitrogen, Darmstadt, Germany, clone RL2). Corresponding Alexa Fluor-conjugated secondary antibodies and the dye HCS CellMask Deep Red Stain (both from Invitrogen) were used for detection of primary antibodies and for cytosol/nucleus staining, respectively. TRAP activity was visualized using a leukocyte acid phosphatase staining kit (Sigma-Aldrich, Steinheim, Germany) with ELF 97 Phosphatase Substrate (Invitrogen) as described^68^. Images and cellomics data were acquired by using a CellInsight CX5 High Content Screening Platform with SpotDetector.V4 BioApplication (Thermo Scientific, Darmstadt, Germany). For cells derived from bone marrow, TRAP^+^F4/80^+^ cells with a single nucleus were identified as immature osteoclasts^69^. TRAP^+^F4/80^−^ cells with more than 3 nuclei were identified as mature osteoclasts. For cells derived from RAW264.7, immature and mature osteoclasts were identified by TRAP activity and nucleus count. Cellomics data were analyzed using the R software (version 3.6.1) with custom codes, and the images were processed by ImageJ software (NIH, version 1.52p).

### Western blot analysis and ProteinSimple Wes immunoassay

Whole cell lysates from in vitro osteoclastogenesis were collected with Cell Lysis Buffer (Cell Signaling Technology, Frankfurt am Main, Germany). Western blots were performed as described previously^70^. Briefly, protein was separated by SDS-PAGE and transferred onto an Immobilon-P membrane (Millipore, Darmstadt, Germany). The membrane was incubated with antibodies against O-GlcNAc (Invitrogen, clone RL2), NFATc1 (Biolegend, clone 7A6), and β-actin (Sigma-Aldrich, clone AC-15) overnight. The blot was visualized with corresponding horseradish peroxidase-conjugated antibodies (Dako, Glostrup, Denmark) and Amersham ECL Prime Western Blotting Detection Reagent (Cytiva, Freiburg, Germany) and a ChemiDoc MP Imaging System (Bio-Rad Laboratories). The images of the bots were analyzed with Image Lab software (Bio-Rad Laboratories, version 6.0.1).

ProteinSimple Wes immunoassay was performed as described^71^ and analyzed by using a Wes system (ProteinSimple, Wiesbaden, Germany) with antibodies against OGT (OriGene Technologies, Herford, Germany), OGA (Sigma-Aldrich), and β-actin.

### Histological and immunofluorescence (IF) analysis of synovial tissue

The histomorphometric analysis was performed as described previously^72^. Hind legs from mice were fixed in 4% formaldehyde and dehydrated in 50% ethanol, followed by decalcification in 500 mmol·L^−1^ acid-free EDTA and paraffin-embedding. Five µm sections of paws were stained with H&E for histology and TRAP for the quantification of the area of bone erosions and osteoclast counts using a leukocyte acid phosphatase staining kit (Sigma-Aldrich). Mature osteoclasts were identified as TRAP-positive multinucleated (nuclei ≥ 3) cells and the immature osteoclasts as TRAP-positive cells with a single nucleus. Images were captured using a Nikon Eclipse 80i microscope (Nikon Metrology, Alzenau, Germany) or a NanoZoomer S60 Digital Slide Scanner (Hamamatsu Photonics, Herrsching am Ammersee, Germany).

The IF analysis was performed as described^73^. Briefly, paw sections were deparaffinized and rehydrated, followed by heat-induced epitope retrieval using boiling 10 mmol·L^−1^ sodium citrate pH 6.0 buffer and Tris-EDTA buffer (10 mmol·L^−1^ Tris, 1 mmol·L^−1^ EDTA, 0.05% Tween-20, pH 9.0). After blocking with 5% horse serum in 2% BSA/PBS, sections were incubated with antibodies against TRAP (Abcam, Berlin, Germany; clone EPR15556 for human tissue; Abnova, Taipei City, Taiwan; polyclonal for mouse tissue), CD14 (Biorbyt, Eching, Germany; polyclonal for human tissue), F4/80 (Bio-Rad Laboratories, clone Cl: A3-1 for mouse tissue), and O-GlcNAc (Invitrogen, clone RL2), followed by alexa fluor-conjugated secondary antibodies (Invitrogen). Confocal images were acquired by using a Leica SP5 II confocal laser scanning microscope (Leica Microsystems, Wetzlar, Germany), and processed with the same contrast adjustment for comparison by using OMERO web platform (University of Dundee & Open Microscopy Environment, version 5.6.3). The quantification of O-GlcNAc in TRAP-positive cells on the articular surface of the tarsus was performed by measuring mean gray value of O-GlcNAc staining within semi-automatically generated region based on TRAP signal using ImageJ software (NIH, version 1.52p) for unbiased quantification.

### Quantitative real-time PCR

Quantification of gene expression by quantitative real-time PCR was performed as described^74^. Briefly, RNA was isolated using a NucleoSpin RNA isolation kit (Macherey-Nagel, Düren, Germany). After reverse transcription, cDNA was mixed with SYBRGreen PCR Master Mix (Applied Biosystems, Darmstadt, Germany) and primer pairs described at Supplementary Table 1. Gene expression was quantified using the comparative CT method with *Actb* as a house keeping gene. Real-time PCR was performed using StepOnePlus Real-Time PCR System (Applied Biosystems). The data were analyzed with StepOne Software v2.3 (Applied Biosystems).

### Quantification of the number of metabolically active cells

The number of metabolically active cells was determined using the Cell Counting Kit-8 (CCK-8, Sigma-Aldrich) following the instruction from the supplier. In brief, the CCK-8 reagent was added to the cells after 3 days of in vitro osteoclastogenesis for 2 h. Then the absorbance at 450 nm was measured by using a GloMax Discover Microplate Reader (Promega, Walldorf, Germany). The number of metabolically active cells was calculated using a standard curve generated by serial dilution of cell number when seeding.

### In vitro bone resorption assay

The bone resorption assays were performed as previously described^75^. In brief, bone marrow cells from mice were seeded on bone slices (Immunodiagnostic Systems, Frankfurt am Main, Germany) with cytokines for in vitro osteoclastogenesis and with or without 20 µmol·L^−1^ OSMI-1 (Sigma-Aldrich) or 10 µmol·L^−1^ Thiamet-G (Tocris, Wiesbaden-Nordenstadt, Germany). The culture medium was changed every 2 days for 2 weeks. The resorption pits were visualized by staining with 1% toluidine blue (Sigma-Aldrich). Images were acquired using a Nikon Eclipse 80i microscope (Nikon Metrology) and then analyzed with ImageJ software (NIH, version 1.52p).

### Arthritis mouse models and in vivo treatments

hTNFα tg mice spontaneously develop chronic inflammatory arthritis^64^. For OGA inhibition, hTNFα tg mice were injected with 6 mg·kg^−1^ Thiamet-G (Tocris) intraperitoneally (i.p.) every other day, starting at the age of four weeks. To knockout *Ogt* in the osteoclast precursors, bone marrow transplantation was performed as described^11^. In brief, four-week-old wildtype or hTNFα tg mice received total body irradiation at the dose of 10.5 Gy. After 24 h, the irradiated mice were transplanted with bone marrow cells from *Ogt*-floxed or *Ogt*^*ΔLysM*^ mice by tail vein injection. The mice were euthanized at the age of nine weeks.

Serum-induced arthritis (SIA) was induced by injection of pooled serum from K/BxN mice as described^72^. Arthritis was initiated in mice at the age of eight weeks by i.p. injection of 200 µL K/BxN serum and boosted with the same amount of serum three days later. To inhibit OGT or OGA, mice were injected i.p. with 10 mg·kg^−1^ OSMI-1 (Sigma-Aldrich) or 6 mg·kg^−1^ Thiamet-G (Tocris), respectively, every other day. Mice were euthanized 14 days after the first serum injection.

Ovariectomy (OVX) -induced osteoporosis was performed by surgical removal of bilateral ovaries^75^. Female *Ogt*-floxed and *Ogt*^Δ*LysM*^ mice were used to evaluate the effect of O-GlcNAc in osteoporosis. OVX and sham operation, in which ovaries were left intact, were performed when the mice reached 8 weeks of age. Mice were kept for five weeks after the operation before euthanization.

The development of arthritis was evaluated by measuring joint swelling at the paws and the grip strength of the mice as described^75^. Bone tissues from the hind legs of the mice were harvested for further analysis. All studies were approved by the government of Unterfranken, Germany.

### Microcomputed tomography (µCT) and analysis

µCT analysis was performed as previously described^76^. Bones of tibiae and paws from mice were preserved in 50% ethanol. µCT images were acquired using a SCANCO Medical μCT 40 scanner (SCANCO Medical AG, Brüttisellen, Switzerland) with optimized settings for visualization of calcified tissue at 55 kVp at a current of 145 µA and 200 ms integration time for 500 projections/180°. The trabecular measurements were performed at a 1 680 µm region located around 400 µm below the middle of the tibia metaphysis. The volume segmentation for the microarchitectural quantification of the trabecular bone was performed using the SCANCO Evaluation Software (SCANCO Medical AG) with a voxel size of 8.4 µm.

### RNA sequencing

RNA sequencing was performed by Novogene Co., Ltd (Cambridge, United Kingdom) on an Illumina NovaSeq platform using a paired-end 150 bp sequencing strategy. Quality assessment and gene mapping were also performed by Novogene Co., Ltd (Cambridge, United Kingdom). Briefly, reads pairs in FASTQ format were quality assessed by FastQC v0.11.5 and mapped to murine genome (GRCm38/mm10). On average, 41% of raw reads were uniquely mapped. Uniquely mapped reads were assigned to annotated genes with Tophat software (Johns Hopkins University). Read counts were normalized by trimmed mean of M values (TMM) method. Principal component analysis (PCA) plots, heatmaps, and volcano plots, and bubble plots were generated with *ggplot2*, *pheatmap*, *EnhancedVolcano*^77^ R packages. Differential gene expression analysis was performed by using R package *edgeR* with Quasi-likelihood F-tests for statistical significance and following thresholds for the identification of differential expression genes (DEGs): false discovery rate-corrected *q* value <0.05 and fold change > 1.5 (for Thiamet-G treated cells) or 2 (for OSMI-1 treated cells). DEGs were subsequently used for gene ontology (GO) gene functional analysis with *clusterProfiler* package^78^. Gene ratios of selected GO terms were plotted as bubble plots. Gene set enrichment analysis (GSEA) was performed using the GSEA software version 4.0.3 (Broad Institute) with a threshold of 0.25 for false discovery rate-corrected *q* value^79^. The curated gene sets for GSEA were downloaded from Bader Lab^80^ (release April 01 2020), which contains pathways from GO biological process^81^, Reactome^82^, Panther^83^, NetPath^84^, NCI^85^, MSigDB^86^ (C2, H collections). Gene sets from GSE10500^87^ and a published gene set for osteoclast differentiation^36^ were also included for GSEA analysis.

To identify distinct genes at different stages of osteoclast differentiation, we analyzed the microarray data from the previous study (accession number GSE138324)^34^ by the web tool GEO2R (NCBI). The unique DEGs at different time points of differentiation were identified with the threshold of *P*-value > 0.05 and fold change >1.5. The DEGs from microarray were further matched to our RNA sequencing data and visualized by heatmaps. To compare the transcriptome of osteoclasts with OSMI-1 treatment with those of MYC knockout osteoclasts, we employed the RNA-Seq data created by Bae et al. (accession number SRP096890) and analyzed as described above^38^.

The information of DEGs was submitted to Ingenuity Pathway Analysis (Qiagen) for upstream regulator analysis. To perform the motif enrichment analyses, the upstream sequences of DEGs were retrieved by RSAT tools^88^ then analyzed by *matrix-scan*^89^ and *AME*^90^ with motifs retrieved from JASPAR (2020) and ENCODE (2018-03) databases. Random sequences generated based on the upstream sequences were used as the background model for the motif enrichment analyses.

### Identification of O-GlcNAcylated proteins by mass spectrometry

Mass spectrometry with O-GlcNAcylated protein was performed as described^91^. Briefly, whole cell lysates were collected from RAW264.7 cells treated with RANKL and TNFα. O-GlcNAcylated protein was further enriched by using Protein A/G agarose beads (Santa Cruz Biotechnology) coupled with antibodies against O-GlcNAc (Invitrogen, clone RL2). Following elution with 2× Lämmli buffer, the enriched protein was separated by SDS-PAGE and visualized by InstantBlue Coomassie staining (Abcam). The protein was in-gel digested with trypsin (Roche), extracted with 70% acetonitrile, 0.03% formic acid, lyophilized, and resuspended in 0.1% TFA (Trifluoroacetic acid). NanoLC-ESI-MS/MS experiments were performed on an Ultimate 3000 nano-RSLC (Thermo Fisher Scientific) coupled to a Q-Exactive HF-X mass spectrometer (Thermo Fisher Scientific) using a Nanospray-Flex ion source (Thermo Fisher Scientific). Peptides were concentrated and desalted on a trap column (5 mm × 30 µm, Thermo Fisher Scientific) and separated on a 25 cm × 75 µm nanoEase MZ HSS T3 reversed-phase column (100 Å pore size, 1.8 µm particle size, Waters, USA) operating at constant temperature of 35 °C. Peptides were separated at a flow rate of 300 nL per min with the following gradient profile: 2%–15% solvent B in 37 min, 15%–30% solvent B in 30 min, 30%–45% solvent B in 23 min, 45%–95% solvent B in 20 min and isocratic at 95% solvent B for 15 min. Solvents used were 0.1% formic acid (solvent A) and 0.1% formic acid in acetonitrile/H_2_O (80/20, v/v, solvent B).

The Q Exactive HF-X was operated under the control of XCalibur 4.1.31.9 software. MS spectra (m/z = 300–1 800) were detected in the Orbitrap at a resolution of 60 000 (m/z = 200) using a maximum injection time (MIT) of 100 ms and an automatic gain control (AGC) value of 1 × 10^6^. Internal calibration of the Orbitrap analyzer was performed using lock-mass ions from ambient air as described^92^. The 30 most abundant peptide precursor signals per MS scan were selected for MS/MS analysis. Peptides were fragmented using high energy collision dissociation (HCD) at a normalized collision energy of 27 and subsequently analyzed in the Orbitrap at a resolution of 15 000. Further settings for MS/MS spectra included an isolation width of 1.6 Da, a MIT of 100 ms and an AGC value of 5 × 10^5^. The dynamic exclusion was set to 20 s.

The protein was identified using the Mascot 2.6.1 software (Matrix Science, London, UK). Spectra were searched against the mouse reference proteome sequence downloaded in FASTA-format from UniProt^93^. Search parameters specified trypsin as cleaving enzyme with three accepted missed cleavages, a 5 ppm mass tolerance for peptide precursors, and 0.02 Da for fragment ions. Carbamidomethylation of cysteine residues was defined as fixed modification. Methionine oxidation, S, T, Y phosphorylation, and O-HexNAc-glycosylation at S and T were considered as variable modifications. Quantitative analysis was done by Scaffold 4.10 software (Proteome Software, Protland, USA) with a threshold of peptide probability >65% and a spectrum counting method. Selected O-glycopeptides were further inspected manually. Neutral loss *N*-acetylglucosamine and the O-GlcNAc oxonium ion (m/z = 204.086) was used as O-GlcNAc diagnostic marker.

### Determine O-GlcNAc levels on NUP153 by immunoprecipitation

Whole cell lysates from in vitro osteoclastogenesis were collected in Cell Lysis Buffer (Cell Signaling Technology) either two (early) or four (late) days after first RANKL stimulation. Following pre-clearing, 50 µg of the lysates were incubated with 0.5 µg antibodies against NUP153 (Santa Cruz Biotechnology, clone R3G1) or NFATc1 (BioLegend, clone 7A6) and pulled down by Protein A/G agarose beads (Santa Santa Cruz Biotechnology). After elution with 2× Lämmli buffer, O-GlcNAc levels were determined by Western blot. Images were analyzed by Image Lab software (Bio-Rad Laboratories, version 6.0.1) with levels normalized to MCSF-treated cells at individual time points.

### Assessment of nuclear accumulation of MYC in osteoclast precursors

RAW264.7 cells were transfected with *Nup153* siRNA and costimulated with RANKL and TNFα as described. To determine the level of chromatin-bound MYC, lysate was collected 2 days after the stimulation. The chromatin-bound faction was obtained using a Subcellular Protein Fractionation kit (Thermo Fisher Scientific) and subjected to Western blot analysis.

The nuclear levels of MYC were evaluated by immunofluorescence staining using anti-MYC (Cell Signaling Technology, clone D84C12), anti-NUP153 (Santa Cruz Biotechnology), and DAPI. Images were acquired using CellInsight CX5 High Content Screening Platform with Colocalization.V4 BioApplication (Thermo Fisher Scientific) for cellomics analysis and Leica SP5 II confocal laser scanning microscope (Leica Microsystems) for confocal microscopy. Nuclear volume was identified from the confocal images by an ImageJ plugin *Colocalization Image Creator* with Otsu thresholding on DAPI signal. The nuclear MYC and perinuclear NUP153 was quantified by ImageJ plugin *MorphoLibJ*^94,95^.

### RNAi and osteoclastogenesis on RAW264.7

*Nup153* knockdown was achieved by transfecting *Nup153* siRNA (Thermo Fisher Scientific) with Lipofectamine RNAiMAX (Thermo Fisher Scientific) reagent following the manufacturer’s instruction. RAW264.7 cells were incubated overnight for post-transfection recovery. Cells were treated with 100 ng·mL^−1^ RANKL (Peprotech) and 20 ng·mL^−1^ TNFα (R&D Systems) to trigger osteoclastogenesis. The culture medium was changed every 2 days. Mature osteoclasts could be found after 4 days of RANKL stimulation.

### Regulation of *Ogt* and *Oga* upon RANKL and TNFα costimulation

To determine the initial response triggered by osteoclastogenic stimulation, bone marrow-derived cells were pre-treated with 1 µmol·L^−1^ SB202190 (Tocris), 5 µmol·L^−1^ of FR180204 (Tocris), 1 µmol·L^−1^ SU6656 (Selleck Chemicals), 40 µmol·L^−1^ T-5224 (ApexBio Technology), or vehicle 2 hours before osteoclastogenic stimulation. Cells were lysed after 6 hours of RANKL + TNFα costimulation, and the *Ogt* and *Oga* mRNA levels were measured by quantitative real-time PCR.

### Statistics

The results are presented as median ± interquartile range (IQR), if not stated elsewhere. The sample sizes (n) are shown in the figure legends. Quantification of cellomics data is shown as violin-histograms, in which the violin graph shows the data distribution within the group, and the histogram shows the proportion of the data from the whole dataset. Mann–Whitney U-tests were applied for the comparison between two groups. ANOVA was applied for multiple comparisons within more than two groups. The post hoc analysis was performed with the Bonferroni method. *P*-values lower than 0.05 were considered statistical significant. All graphs and statistics were generated using Prism9 (GraphPad Software) and the R software (version 3.6.1) with *tidyverse* and *see* packages.

### Study approval

This study was approved by the ethical committee of the University of Erlangen-Nürnberg. All patients recruited in this study signed informed consent. The animal experiments were approved by the regional government (Regierung von Unterfranken, Würzburg, Germany).

## Supplementary information

Supplementary files

## Data Availability

The information of data generated and analyzed during this study are available from the corresponding author on request.
