# Supplementary material for: Dynamic changes in O-GlcNAcylation regulate osteoclast differentiation and bone loss via nucleoporin 153
Source: Bone Res. 2022 Jul 26;10:51. doi: 10.1038/s41413-022-00218-9 (PMC9314416; doi:10.1038/s41413-022-00218-9)
Supplement: Supplementary file 1 — Supplementary files [file 41413_2022_218_MOESM1_ESM.docx]

**Supplementary Information**

**Dynamic changes in O-GlcNAcylation regulate osteoclast differentiation and bone loss via nucleoporin 153**

**Authors:** Yi-Nan Li, Chih-Wei Chen, Thuong Trinh-Minh, Honglin Zhu, Alexandru-Emil Matei, Andrea-Hermina Györfi, Frederic Kuwert, Philipp Hubel, Xiao Ding, Cuong Tran Manh, Xiaohan Xu, Christoph Liebel, Vladyslav Fedorchenko, Ruifang Liang, Kaiyue Huang, Jens Pfannstiel, Min-Chuan Huang, Neng-Yu Lin, Andreas Ramming, Georg Schett, Jörg H.W. Distler^*^

*Corresponding author: Jörg H. W. Distler, MD; Department of Internal Medicine 3 – Rheumatology and Immunology, Friedrich Alexander University Erlangen-Nuremberg (FAU) and Universitaetsklinikum Erlangen, Ulmenweg 18, 91054 Erlangen, Germany, Tel.: +49 9131 43008, FAX: +49 9131 35467, Email: joerg.distler@uk-erlangen.de

**Supplementary table**

**Supplementary Table 1. Primer pairs used to determine mRNA levels by qPCR**

| Primer pairs | Forward primer (5’–3’) | Reverse primer (5’–3’) |
| --- | --- | --- |
| *Actb* | TCTTTGATGTCACGCACGAT | ACAGCTTCACCACCACA |
| *Acp5* | CGACCATTGTTAGCCACATACG | TCGTCCTGAAGATACTGCAGGTT |
| *Nfatc1* | CAACAAGCGCAAGTACAGTCTC | CAGGTATCTTCGGTCACACTGA |
| *Ogt* | GCACAAGGGGAGATTTGGCT | GCGTGCCTCTTTCAAGACATT |
| *Oga* | CGGGGGCTTCGTTGGAGCAG | GTTATCTTCTCCGGGGGCGGG |

**Supplementary figures**

**
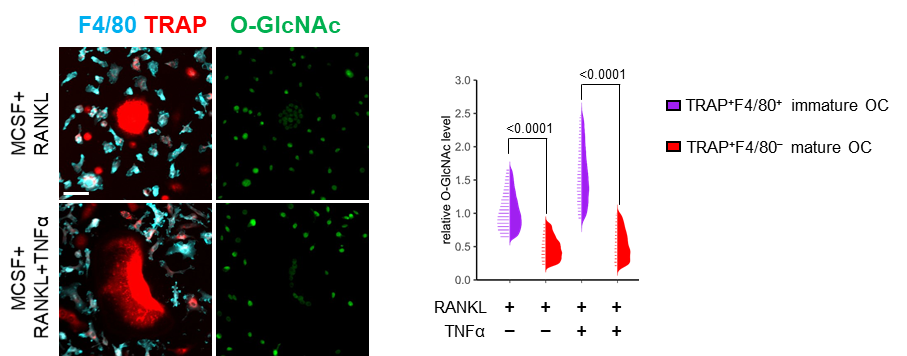
**

**Supplementary Fig. 1 Cellomics analysis of O-GlcNAcylation levels in osteoclasts at different stages of maturation.**

Representative images of high-content imaging and cellomics analysis for O-GlcNAcylation levels in RANKL and TNFα treated osteoclasts at immature and mature stages (n = 30019 cells in total). Horizontal scale bar represents 50 µm. Statistical significance was determined by two-way ANOVA. *P*-values are shown in the graphs. OC, osteoclast.

**
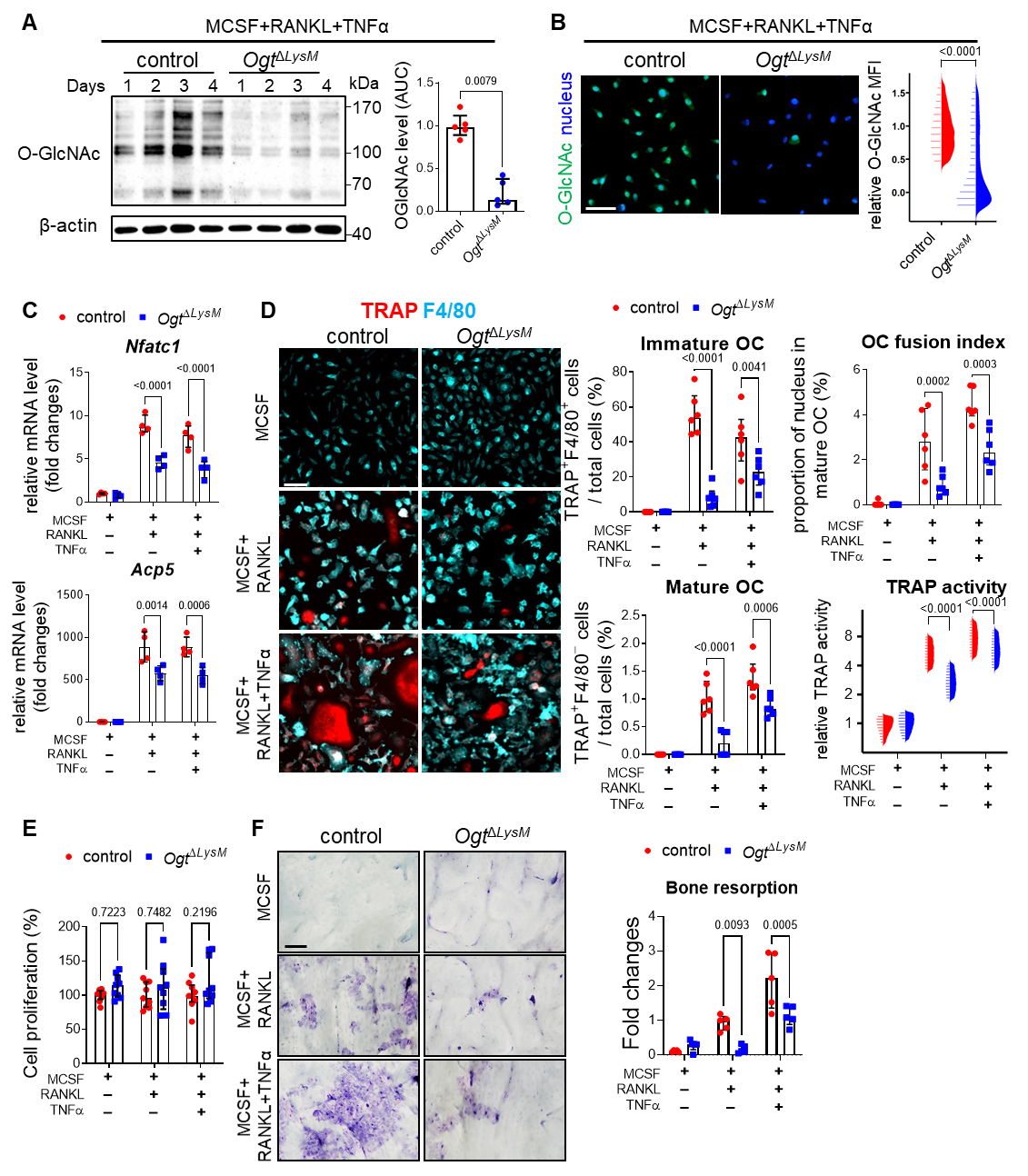
**

**Supplementary Fig. 2 Knockout of OGT knockout inhibits osteoclastogenesis.**

(**A and B**) Western blot analysis (**A**), representative images of high-content imaging, and cellomics analysis (**B**) of O-GlcNAcylation during *in vitro* osteoclastogenesis using bone marrow cells from *Ogt^∆LysM^* mice and control littermates (n = 5 per group for Western blot; n = 30914 cells in total). (**C**) mRNA levels of osteoclast-related genes (n = 4 per group). (**D**) Representative images of high-content imaging and cellomics analysis of the proportions of immature and mature OC, OC fusion index, and TRAP enzyme activity (n = 204782 cells in total). (**E**) Proliferation assay (n = 9 per group). (**F**) *in vitro* bone resorption assay using bone marrow cells from *Ogt^∆LysM^* mice and control littermates (n = 5 per group).

All bar graphs are shown as median ± IQR. Horizontal scale bars are represented as 50 µm in IF images, 100 µm in bone resorption assay. Statistical significance was determined by Mann-Witney U-test (**A and B**) or two-way ANOVA (**C to F**). *P*-values are shown in the graphs. AUC, area under the curve; MFI, mean fluorescence intensity, OC, osteoclast.


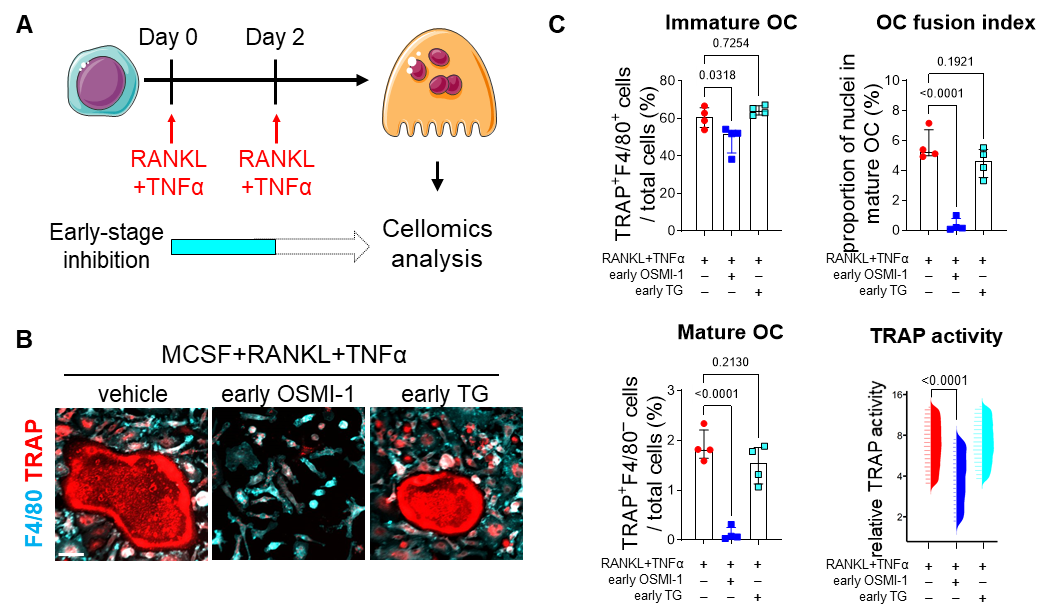


**Supplementary Fig. 3 Early-stage inhibition of OGT, but not OGA, suppresses osteoclast formation.**

**(A**) Schematic diagram of inhibitor treatment for early-stage (Day0 to Day2) inhibition. The diagram was created by adapting images from Servier Medical Art (<http://smart.servier.com/>) under CC BY 3.0 (<https://creativecommons.org/licenses/by/3.0/>) license. (**B** **and** **C**) Representative images of high-content imaging (**B**) and cellomics analysis of proportions of immature and mature OC, OC fusion index, and TRAP enzyme activity (**C**) on osteoclasts treated with vehicle, OSMI-1, and Thiamet-G (TG) at early stages (Day0 to Day2; n = 33304 cells in total).

All bar graphs are shown as median ± IQR. Horizontal scale bars are represented as 50 Statistical significance was determined by one-way ANOVA. *P*-values are shown in the graphs. OC, osteoclast.


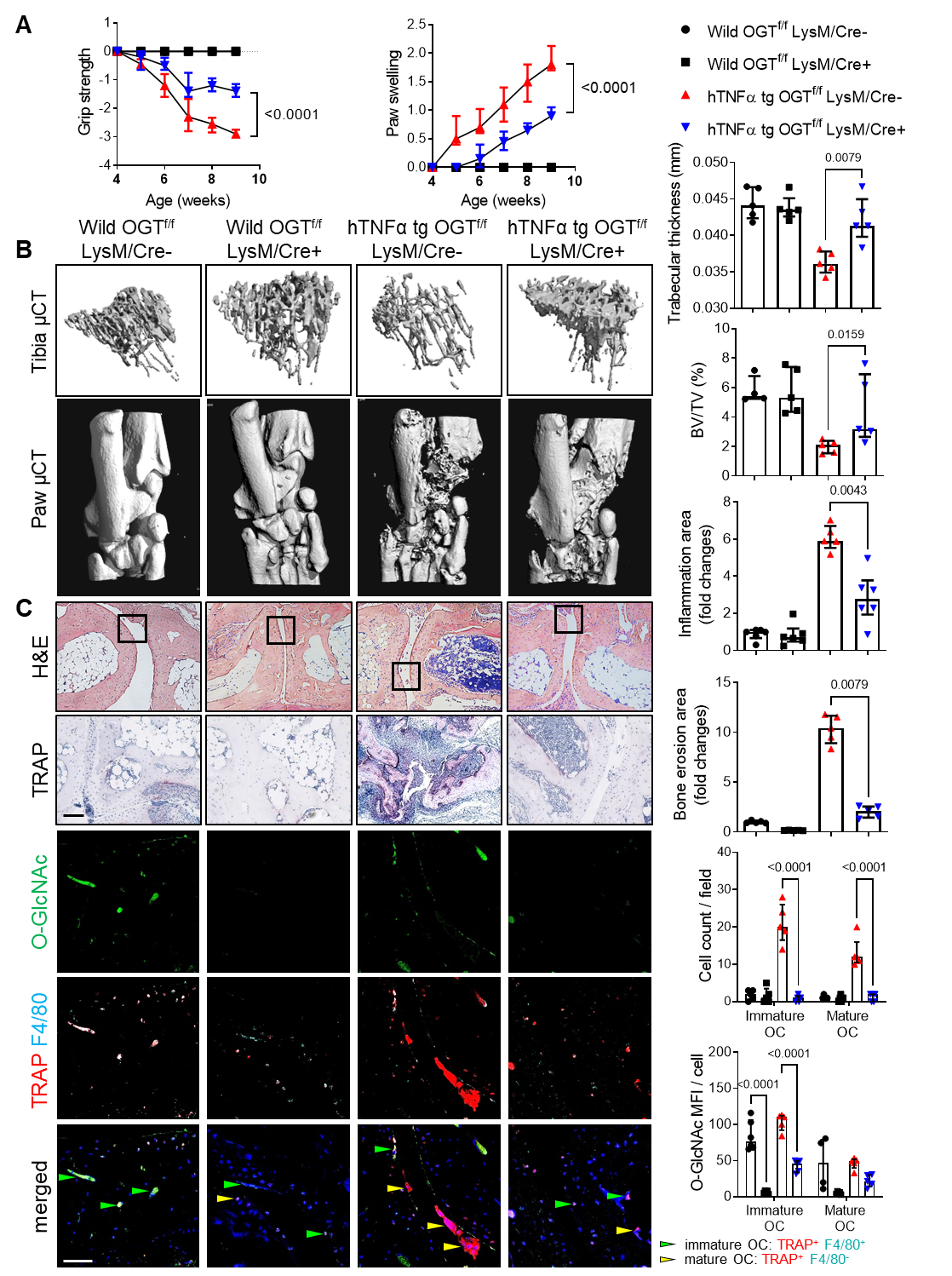


**Supplementary Fig. 4 Knockout of OGT ameliorates bone loss in human TNFα transgene mice.**

(**A**) Grip strength and joint swelling in hTNFα tg mice transplanted with bone marrow from *Ogt^∆LysM^* mice and control littermates. (**B)** Images and quantification of microcomputed tomography scans of bone tissue and analysis of the tibial bone structure. (**C**) Representative histological and cofocal images of the tarsus with semi-automated, blinded quantification of H&E staining, TRAP staining, and O-GlcNAcylation. (n ≥ 5 for all groups)

All results are presented as median ± IQR. Horizontal scale bars are represented as 50 µm in IF images, 100 µm in histological staining. Statistical significance was determined by two-way ANOVA (**A and C**) or Mann-Whitney U-test (**B)**. *P*-values are shown in the graphs. MFI, mean fluorescence intensity, OC, osteoclast.


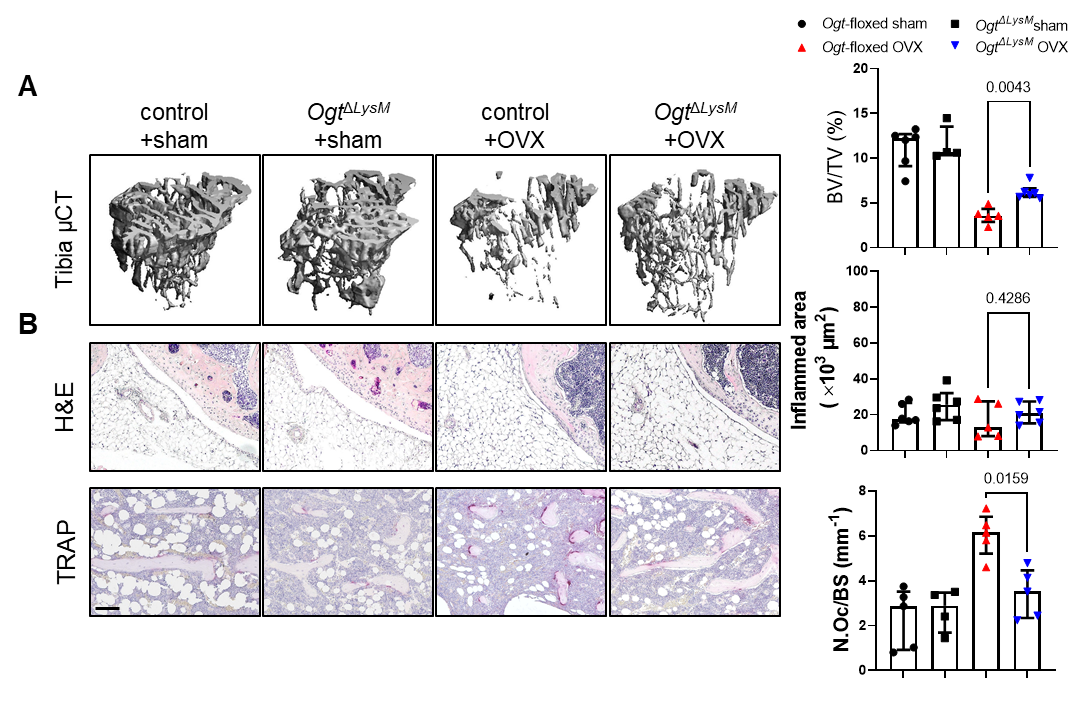


**Supplementary Fig. 5 Knockout of OGT mitigates bone loss in ovariectomy-induced osteoporosis.**

**(A**) Images and quantification of microcomputed tomography scans of bone tissue and the analysis of the tibial bone structure. (**B**) Representative histological images with semi-automated, blinded quantification of H&E staining and TRAP staining. (n ≥ 5 for all groups)

All results are presented as median ± IQR. Horizontal scale bars are represented as 100 µm in histological staining. Statistical significance was determined by Mann-Whitney U-test. *P*-values shown in the graphs. N.Oc, number of osteoclast.

**
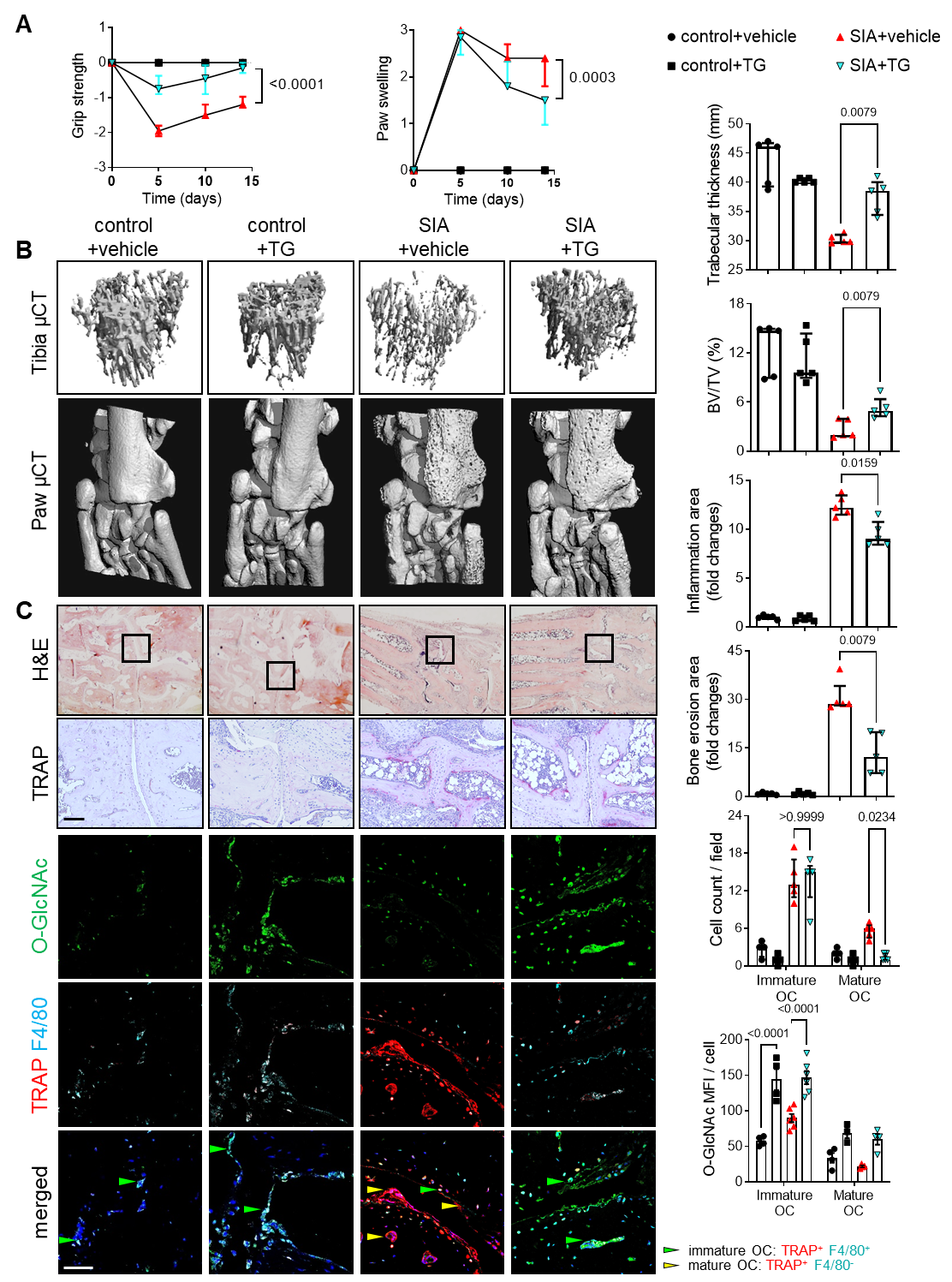
**

**Supplementary Fig. 6 Pharmacological inhibition of OGA ameliorates bone loss in serum-induced arthritis.**

(**A**) Grip strength and joint swelling. (**B)** Images and quantification of microcomputed tomography scans of bone tissue and the analysis of the tibial bone structure. (**C**) Representative histological and confocal images with semi-automated, blinded quantification of H&E staining, TRAP staining, and O-GlcNAcylation. (n ≥ 5 for all groups)

All results are presented as median ± IQR. Horizontal scale bars are represented as 50 µm in IF images, 100 µm in histological staining. Statistical significance was determined by two-way ANOVA (**A and C**) or Mann-Whitney U-test (**B)**. *P*-values shown in the graphs. MFI, mean fluorescence intensity, OC, osteoclast.


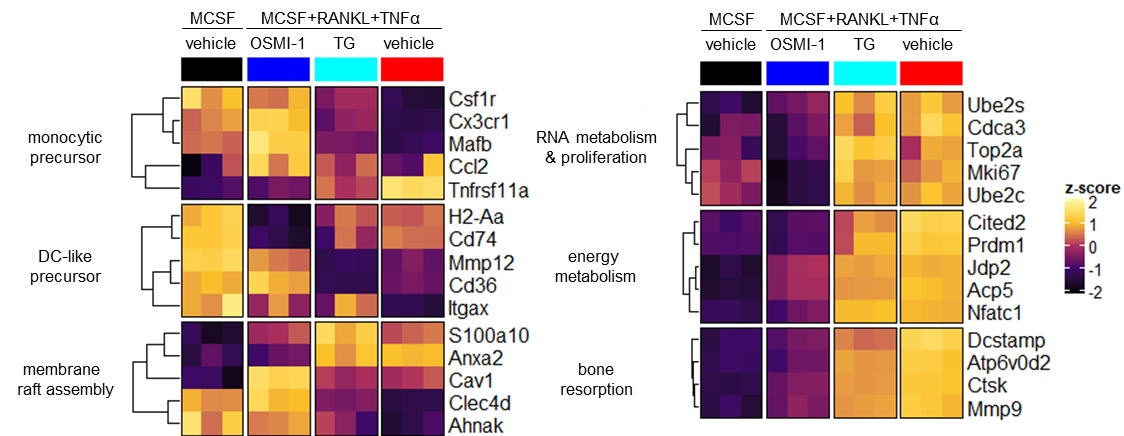


**Supplementary Fig. 7 OGT and OGA inhibition arrest cells at different differentiation states.**

Expression heatmap of previous published signature genes for each functional stage during osteoclastogenesis^1^ on cells treated with OSMI-1 and Thiamet-G (TG), demonstrating stage-specific arrest at early or late stages of osteoclast differentiation with OSMI-1 and TG, respectively.

**
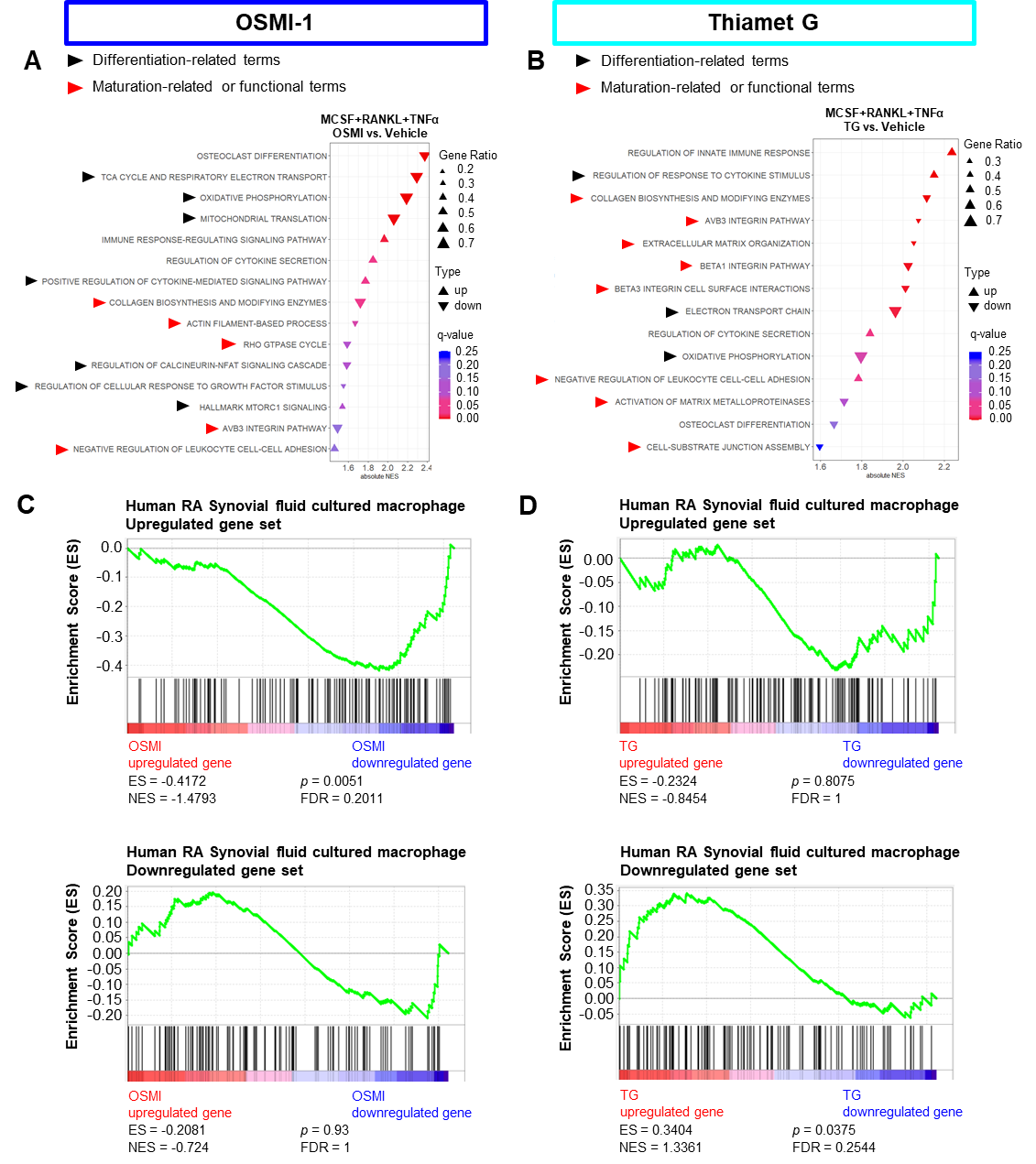
**

**Supplementary Fig. 8 Differential transcriptional changes of OSMI-1 and Thiamet-G in osteoclast precursors.**

(**A and B**) Plots of significantly enriched gene sets related to osteoclastogenesis in response to treatment with OSMI-1 (**A**) or Thiamet-G (**B**) by GSEA. (**C and D**) GSEA enrichment plots for genes up- or downregulated in human macrophages obtained from synovial fluid of inflamed joints of patients with RA (GSE10500) ^2^ for OSMI-1 (**C**) and Thiamet-G (**D**) treated cells.

**
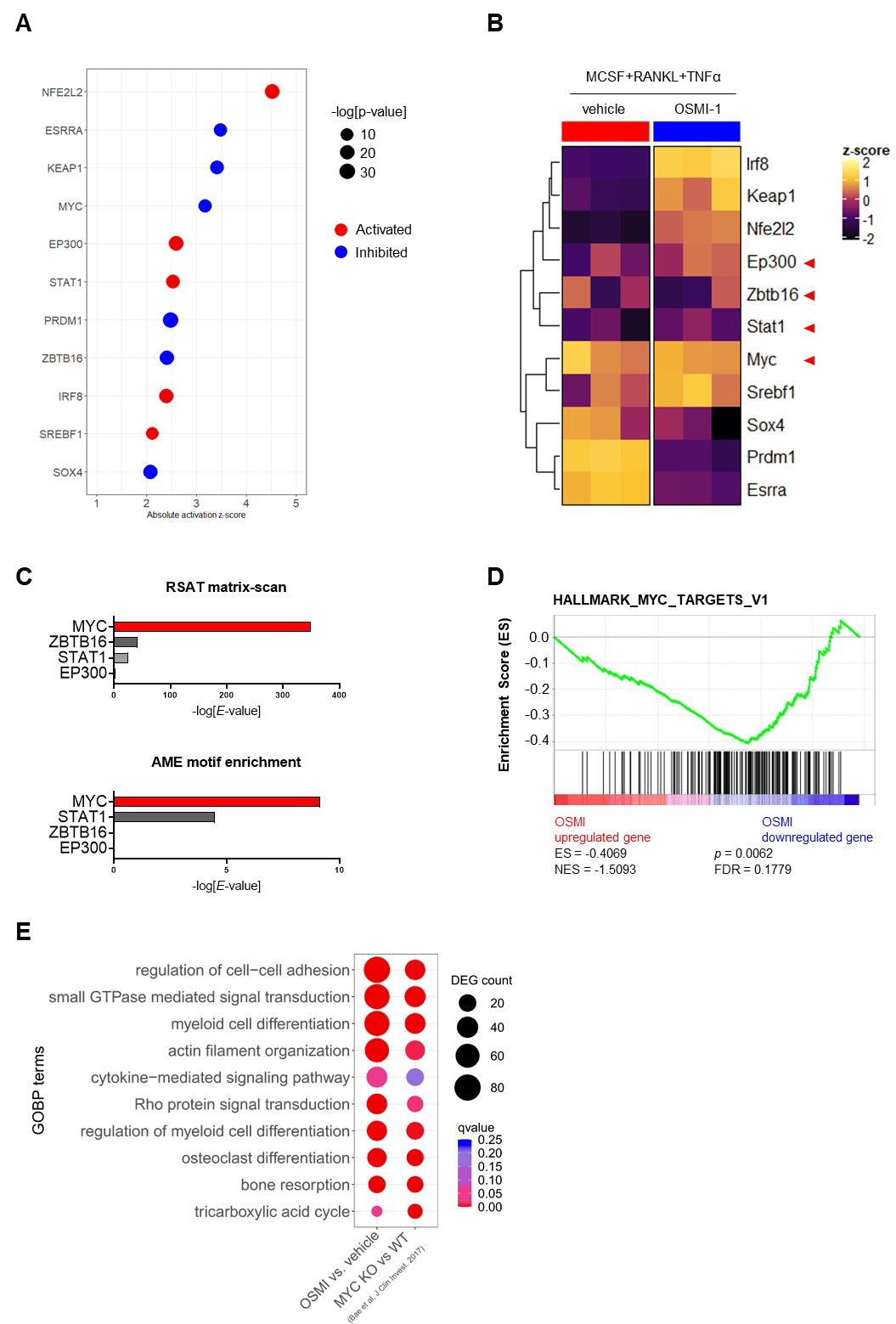
**

**Supplementary Fig. 9 Bioinformatic analyses of IPA predicted transcription regulators.**

(**A**) Potential upstream transcriptional regulators of the DEGs in OSMI-1 treated cells using IPA. (**B**) Expression heatmap of the respective transcription regulators. The genes without significant changes in mRNA levels are highlighted with red arrows. (**C**) Enrichment analyses for the motifs of transcription regulators in DEG upstream sequences. (**D**) GSEA enrichment plot of genes regulated by MYC for OSMI-1 treated osteoclast precursors. (**E**) Geneset functional analysis plots for GO biological process terms that overlap between OSMI-1 treated osteoclasts and osteoclasts with MYC knockout (accession number SRP096890)^3^.

**
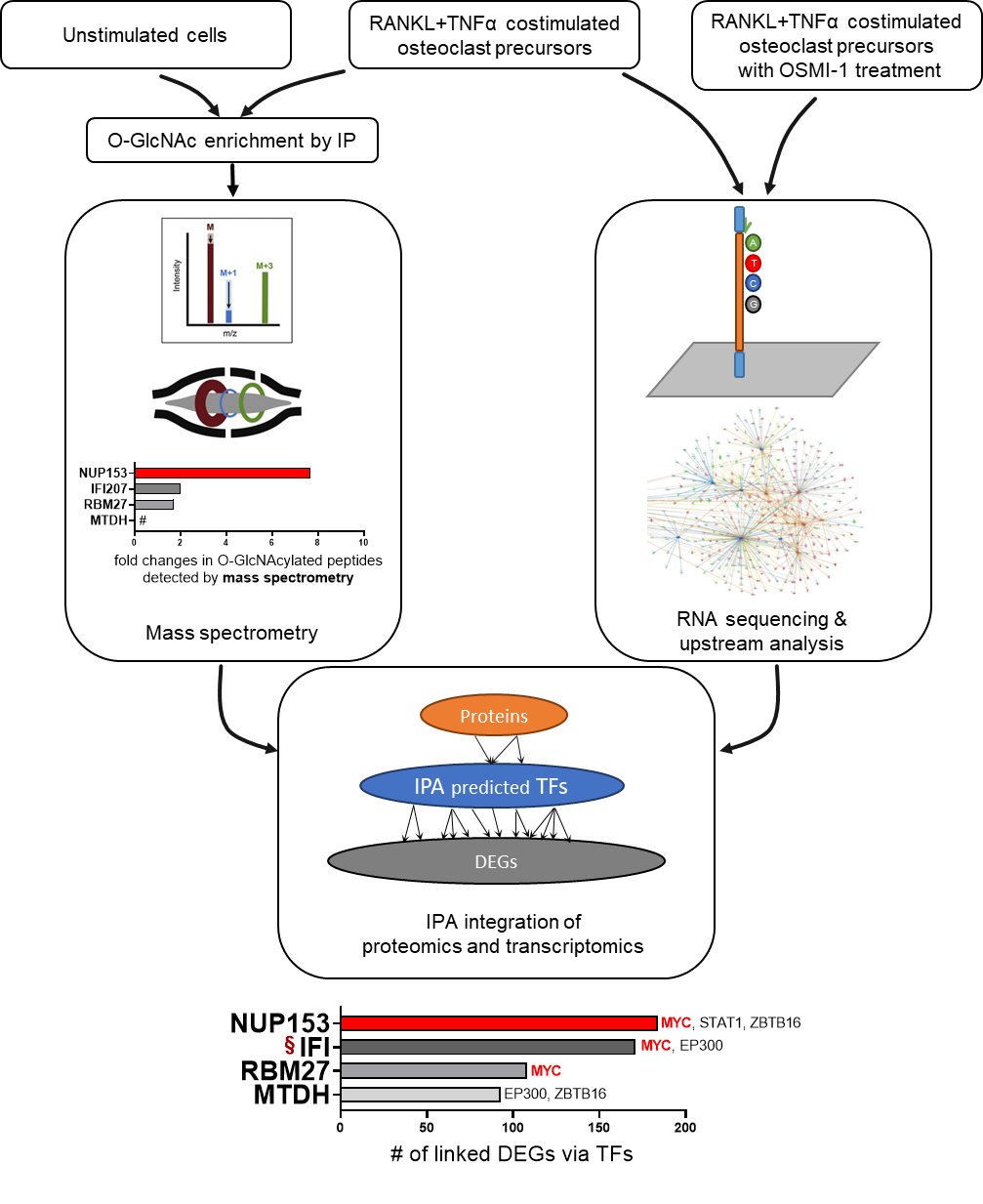
**

**Supplementary Fig. 10 Schematic workflow for O-GlcNAc target screening.**

Following enrichment of O-GlcNAc modified protein by immunoprecipitation, proteins with increased O-GlcNAc levels in RANKL+TNFα costimulated cells compared to unstimulated cells were detected by mass spectrometry and taken for further analysis. To evaluate the potential of these targets to regulate O-GlcNAc-dependent transcriptome, we retrieved the interactions between the targets and the predicted transcription regulators using IPA. The linked transcription regulators and the number of corresponding DEGs from the RNAseq dataset obtained from OSMI-1 treated osteoclast precursors are shown in the graph.

#, unable to compute fold change since O-GlcNAc modified MTDH was not detected in unstimulated cells.

§, the Ingenuity Knowledge Base curates several IFIs as one target; specific IFI207 targets are not defined. The effect of IFI207 on DEGs might thus be overestimated.

The diagram of mass spectrometry is adapted from Hofmann et al.^4^ under CC BY 4.0 license (<https://creativecommons.org/licenses/by/4.0/>).

**
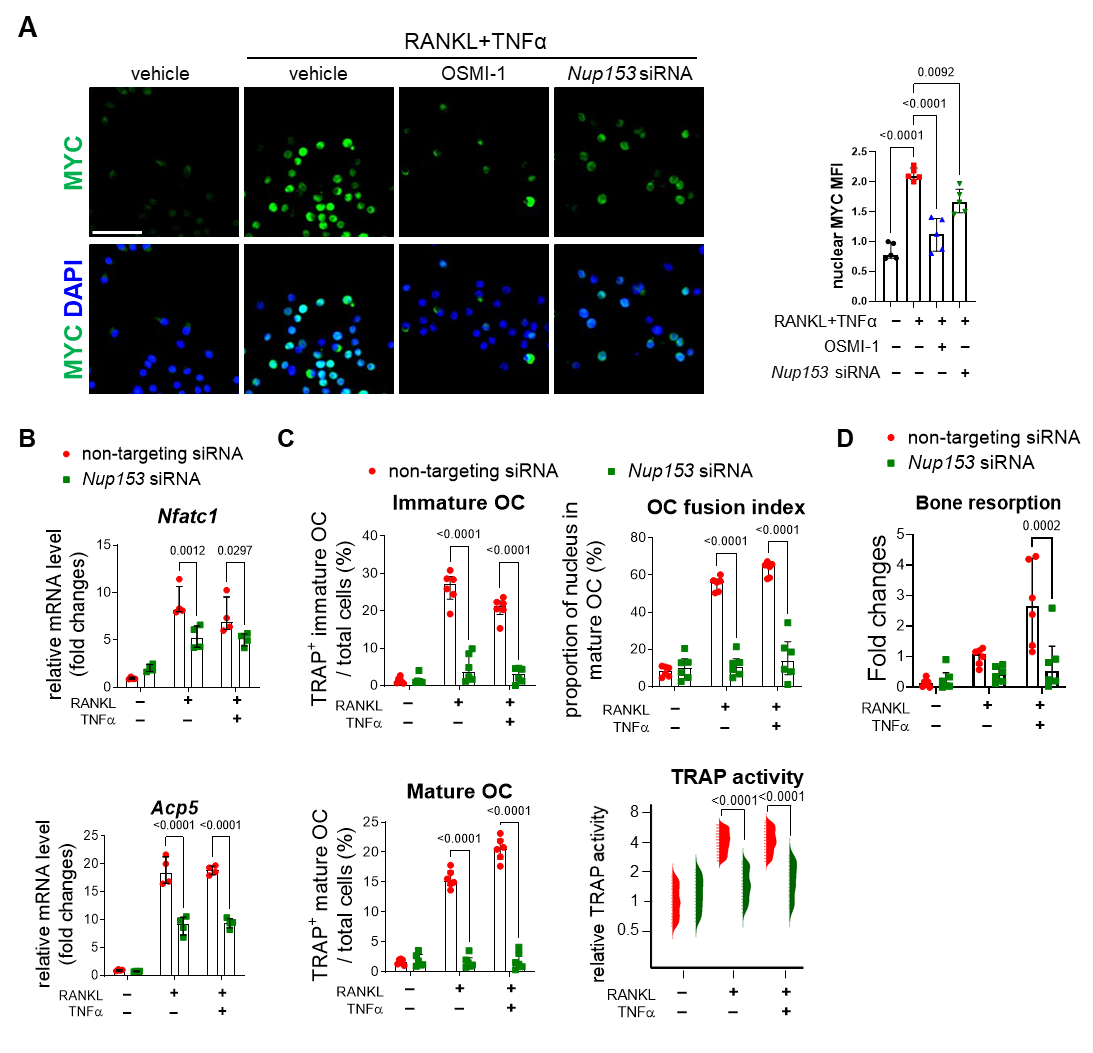
**

**Supplementary Fig. 11 *Nup153* knockdown inhibits MYC nuclear co-localization and osteoclastogenesis.**

(**A**) Representative immunofluorescence image and cellomics analysis of MYC nuclear co-localization on RAW264.7 cells transfected with *Nup153* siRNA (n = 5 replicates per group; >1000 cells analyzed for each replicate). Unless specified, cells were transfected with non-targeting siRNA and treated with vehicle. Horizontal scale bar, 50 µm. (**B**) Osteoclast-related gene expression in cells with *Nup153* knockdown (n = 4 per group). (**C**) Cellomics analysis of proportions of immature and mature osteoclasts, OC fusion index, and TRAP enzyme activity in cells with *Nup153* knockdown (n = 34855 cells in total). (**D**) *In vitro* bone resorption assay on cells with *Nup153* knockdown (n = 6 per group). MFI, mean fluorescence intensity; OC, osteoclast.

**References**

1. Tsukasaki, M.*, et al.* Stepwise cell fate decision pathways during osteoclastogenesis at single-cell resolution. *Nat Metab* **2**, 1382-1390 (2020).

2. Yarilina, A., Park-Min, K.H., Antoniv, T., Hu, X. & Ivashkiv, L.B. TNF activates an IRF1-dependent autocrine loop leading to sustained expression of chemokines and STAT1-dependent type I interferon-response genes. *Nat Immunol* **9**, 378-387 (2008).

3. Bae, S.*, et al.* MYC-dependent oxidative metabolism regulates osteoclastogenesis via nuclear receptor ERRalpha. *J Clin Invest* **127**, 2555-2568 (2017).

4. Hofmann, A.E.*, et al.* Using Orbitrap mass spectrometry to assess the isotopic compositions of individual compounds in mixtures. *International Journal of Mass Spectrometry* **457**, 116410 (2020).
